# Decoding the Heterogeneity of Diffuse Large B‐Cell Lymphomas: A Comprehensive Genetic, Transcriptomic, and Phenotypic Profiling of B‐Cell Lymphoma Cell Lines

**DOI:** 10.1002/hon.70228

**Published:** 2026-08-05

**Authors:** Marina Pérez‐Aguilera, Lucía Pedrosa, Sagrario Gómez, Sara Salazar‐Ortego, Ismael Fernández‐Miranda, Rafael Muñoz‐Viana, Paloma Martín‐Acosta, Beatriz L. Martín‐Lunas, Rebeca Jimeno, Natalia Yanguas‐Casás, Beatriz Horcajo, Giovana Roncador, José A. Martínez‐Climent, Ana Ortega‐Molina, Margarita Sánchez‐Beato

**Affiliations:** ^1^ Medical Oncology Department Lymphoma Research Group Hospital Universitario Puerta de Hierro‐Majadahonda IDIPHISA Madrid Spain; ^2^ Immune System Development and Function Department Metabolism in Cancer and Aging Research Group Centro de Biologia Molecular Severo Ochoa (CBM), CSIC—Universidad Autonoma de Madrid Madrid Spain; ^3^ Bioinformatics Unit Hospital Universitario Puerta de Hierro‐Majadahonda IDIPHISA Madrid Spain; ^4^ Pathology Department Cancer Molecular Pathology Group Hospital Puerta de Hierro‐Majadahonda IDIPHISA Madrid Spain; ^5^ Centro de Investigación Biomédica en Red de Fragilidad y Envejecimiento Saludable (CIBERFES) Madrid Spain; ^6^ Monoclonal Antibodies Core Unit, Biotechnology Program, Spanish National Cancer Research Centre (CNIO) Madrid Spain; ^7^ Department of Haematology Center for Applied Medical Research Cancer Center Clinica Universidad de Navarra IDISNA CIBERONC Pamplona Spain

**Keywords:** cell lines, diffuse large B‐cell lymphoma, genetic subtypes, high‐grade, molecular classification, transcriptomics

## Abstract

Diffuse large B‐cell lymphoma (DLBCL) is the most prevalent form of non‐Hodgkin lymphoma, exhibiting significant molecular and clinical heterogeneity. Advances in classification integrating phenotypic, genetic, and transcriptomic features have improved diagnosis and prognosis. However, a comprehensive and integrated molecular characterization of DLBCL cell lines is still lacking, which limits their optimal use as reliable experimental models. We employed fluorescence in situ hybridization, immunohistochemistry, and targeted DNA and RNA sequencing to identify genetic subtypes and determine the cell of origin, providing a comprehensive characterization of 29 DLBCL cell lines through the integration of phenotypic, genomic, and transcriptomic data. Principal component analysis, gene set enrichment analysis (GSEA), differential expression profiling, and regulon analysis enabled us to dissect molecular heterogeneity. We achieved high concordance in genetic subtype assignment using multiple classification algorithms (2‐S, LymphGen, and DLBclass). The DHIT/DZ signature and transcriptional profiling further revealed additional molecular complexity. Some DLBCL‐NOS cases exhibited high‐grade features, suggesting that gene expression signatures may capture biological aggressiveness better than cytogenetic methods. GSEA confirmed the relevance of signaling pathways across DLBCL subtypes, and regulatory network analysis identified specific transcription‐factor activities that support these pathways. MCD cell lines showed increased NF‐κB and STAT signaling, while EZB/MYC+ cell lines demonstrated increased proliferation and cell cycle regulation, along with decreased NF‐κB/STAT activity. Our study offers a detailed molecular overview of DLBCL cell lines, underscoring their relevance for mechanistic and therapeutic research. The data highlights how integrating genetic and transcriptomic analyses can refine disease classification and guide personalized therapy strategies.

## 1 Introduction

Diffuse large B‐cell lymphoma (DLBCL), the most common type of non‐Hodgkin lymphoma (NHL), is an aggressive and heterogeneous disease. The World Health Organization (WHO) Classification of Tumors of Hematopoietic and Lymphoid Tissues (WHO‐HAEM4) classifies DLBCL into morphological variants, molecular subtypes, and distinct entities, with unclassified cases designated as DLBCL‐NOS (not otherwise specified) [1]. Gene‐expression profiling stratified DLBCL by cell‐of‐origin (COO) into germinal center B‐cell (GCB), activated B‐cell (ABC), and unclassified subtypes [2, 3], with ABC‐DLBCL associated with a poorer prognosis [2, 3, 4, 5].

WHO‐HAEM4 included two categories of high‐grade B‐cell lymphoma (HGBL): HGBL with *MYC* and *BCL2* and/or *BCL6* rearrangements (double‐hit [DH] or triple‐hit [TH]), and HGBL‐NOS [1, 6]. In WHO‐HAEM5 (2022) [7], cases with *MYC* and *BCL6* rearrangements were classified as DLBCL‐NOS or HGBL‐NOS depending on morphology. In contrast, the 2022 International Consensus Classification (ICC) [8] maintained HGBL‐DH‐*BCL2* and introduced HGBL‐DH‐*BCL6* as a provisional entity [8, 9]. Gene‐expression studies identified biologically distinct signatures, including the double‐hit expression signature (DHITsig) [10], resembling HGBL‐DH‐*BCL2*, and the dark‐zone signature (DZsig) [11] observed even in cases without *MYC*/*BCL2* rearrangements. A molecular high‐grade (MHG) class overlapping with HGBL‐DH‐*BCL2* was also identified [12].

Large‐scale genomic profiling has further refined DLBCL taxonomy. Schmitz et al. [13] and Wright et al. [14] identified six genetic profiles: MCD, BN2, and N1, predominantly ABC‐DLBCL; ST2 and EZB, mostly GCB‐DLBCL; and A53, enriched in cases with high aneuploidy and *TP53* mutations. Chapuy et al. [15, 16] identified five subtypes: two ABC‐DLBCL groups (C1, low‐risk, resembling BN2; and C5, high‐risk, similar to MCD), two GCB‐DLBCL subgroups (C4, similar to ST2, with a favorable prognosis, and C3, with a poorer prognosis, similar to EZB); and C2, an aneuploidy‐rich, COO‐independent subtype (similar to A53). Lacy et al. [17] confirmed this heterogeneity in a real‐world DLBCL cohort, identifying BCL2 (EZB/C3‐like), SOCS1/SGK1 and TET2/SGK1 (ST2/C4‐like), MYD88 (MCD/C5‐like), and NOTCH2 (BN2/C1‐like) subtypes [17, 18]. Our group validated these classifications and proposed a two‐step (2‐S) algorithm for classifying DLBCL samples into MCD^2−S^, BN2^2−S^, N1^2−S^, EZB^2−S^, and ST2^2−S^, demonstrating strong concordance across the three classification approaches [19].

Despite these advances, preclinical research often relies on cell lines with inconsistently reported molecular features. Ben‐David et al. [20] found that ∼19% of mutations differed between paired cultures of the same lines, while Juskevicius et al. [21] noted discrepancies between newly generated and published profiles [22, 23, 24]. Variations in culture conditions, analytical platforms, and classification criteria contribute to this variability [20, 25, 26, 27], emphasizing the need for standardized characterization.

Our study comprehensively analyzes 29 DLBCL lines using fluorescence in situ hybridization (FISH), immunohistochemistry (IHC), targeted DNA sequencing, and RNA sequencing. Integrating these multimodal data, we refine molecular classifications and provide a robust reference resource to support more precise therapeutic development for distinct DLBCL subtypes.

## 2 Materials and Methods

### 2.1 Cell Lines

Twenty‐nine DLBCL lines were cultured under standard conditions (37°C, 5% CO_2_). Details are provided in Supporting Information S1: Supplemental Methods and Supporting Information S3: Table S1.

### 2.2 Tissue Microarray, Immunohistochemistry and Fluorescence in situ Hybridization

Formalin‐fixed and paraffin‐embedded cell line (FFPEcl) pellets were prepared, and two 1‐mm cores per FFPEcl block were included in a tissue microarray (TMA) using a tissue arrayer (Beecher Instruments Inc.) IHC was performed for COO assessment; FISH was performed for *MYC*, *BCL2*, and *BCL6* rearrangements (details in Supporting Information S1: Supplemental Methods).

### 2.3 DNA‐Sequencing

Genomic DNA was extracted from ∼1.5 × 10^6 cells using the Maxwell 16 Cell LEV DNA Purification Kit and the Maxwell 16 Instrument AS2000 (Promega Biotech Iberica SL, Madrid, Spain) according to the manufacturer's instructions. A custom target‐enrichment panel covering known B‐cell lymphoma driver genes and pathway components was used (Supporting Information S3: Table S2) [19, 28].

### 2.4 RNA‐Sequencing

RNA was extracted from ∼1.5 × 10^6 cells using the Maxwell 16 Cell LEV Total RNA Purification Kit and the Maxwell 16 Instrument AS2000 (Promega Biotech Iberica SL) according to the manufacturer's instructions. Libraries were generated using the QuantSeq 3′ mRNA Seq Library Prep Kit‐FWD (Lexogen GmbH, Vienna, Austria) according to the manufacturer's guidelines. The libraries were sequenced with 75 bp single‐end reads on Illumina NextSeq 550 (Genomics Unit, CNIO, Madrid, Spain).

Information on DNA‐ and RNA‐seq bioinformatic analyses and gene expression analyses is detailed in Supporting Information S1: Supplemental Methods and Supporting Information S2: Figure S1.

## 3 Results

### 3.1 Genetic Characterization of DLBCL Cell Lines

**FIGURE 1:**
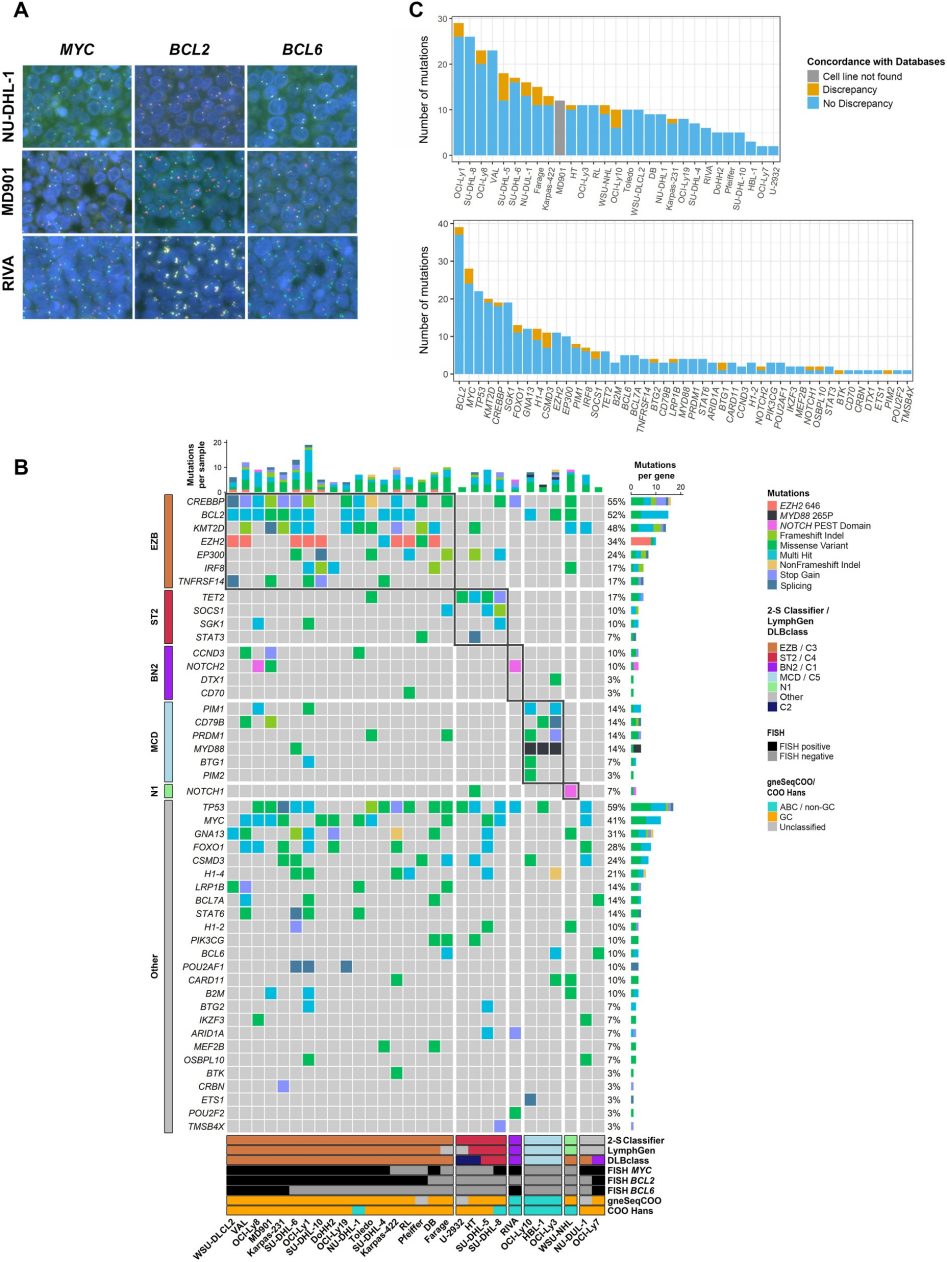
Genetic analysis of diffuse large B‐cell lymphoma‐derived cell lines. (A) Fluorescence in situ hybridization (FISH) images of three selected cell lines: NU‐DHL‐1 (HGBL‐DH‐*BCL2,* with *MYC* and *BCL2* rearrangements), MD901 (HGBL‐DH‐*BCL2* with *BCL6* rearrangement), and RIVA (DLBCL‐NOS, with *MYC* and *BCL6* rearrangements and *BCL2* gain). Images were captured using a 100x objective with mineral oil. Vysis dual‐color break‐apart probes were used to detect the rearrangements. (B) Oncoplot of the 29 cell lines, displaying the mutations identified by DNA‐targeted sequencing, genetic subtypes determined by 2‐S, LymphGen and DLBclass classifiers, as well as FISH results for *MYC*, *BCL2*, and *BCL6,* along with cell‐of‐origin (COO) classifications determined by gneSeqCOO (activated B‐cells [ABC], germinal center B‐cells [GC] or unclassified) and Hans' algorithm (non‐GC or GC). Black squares represent mutations defining the different genetic subgroups. (C) Absolute frequency of discrepancies in detected mutations by DNA‐targeted sequencing compared with external data, analyzed by cell line (top) and by gene (bottom).

We assessed *MYC*, *BCL2*, and *BCL6* rearrangements in 29 DLBCL lines using FISH break‐apart probes, following clinical diagnostic standards. Because some results differed from published reports [29, 30], additional assays were performed: *BCL2* dual‐fusion probes were used for DB, SU‐DHL‐4, and WSU‐NHL, and an alternative *MYC* dual‐break‐apart probe was used for DB, SU‐DHL‐4, and RL. These analyses identified a *BCL2* translocation and a complex *MYC* break involving a centromeric derivative in SU‐DHL‐4, while WSU‐NHL lacked a *BCL2* rearrangement. The DB line yielded no evaluable signals (Supporting Information S3: Table S3). Based on this, 14 lines were classified as HGBL‐DH‐*BCL2*: eight with *MYC* and *BCL2* translocations (DoHH2, NU‐DHL‐1, OCI‐Ly1, OCI‐Ly19, SU‐DHL‐4, SU‐DHL‐6, SU‐DHL‐10 and Toledo), while six also harbored *BCL6* rearrangements (Karpas‐231, MD901, OCI‐Ly7, OCI‐Ly8, VAL and WSU‐DLCL2). RIVA exhibited *MYC* and *BCL6* breaks plus *BCL2* amplification and was categorized as DLBCL‐NOS, though also compatible with the 2022‐ICC provisional HGBL‐DH‐*BCL6* category. The remaining 14 lines (DB, Farage, HBL‐1, HT, Karpas‐422, NU‐DUL‐1, OCI‐Ly3, OCI‐Ly10, Pfeiffer, RL, SU‐DHL‐5, SU‐DHL‐8, U‐2932, and WSU‐NHL) were classified as DLBCL‐NOS (Table 1 and Figure 1A; DLBCL Cell Line Explorer).

**TABLE 1:** Classifications of the diffuse large B‐cell lymphoma‐derived cell lines.

Cell line	DLBCL entity [8]	2‐S [19]	LymphGen [14]	DLBclass [16]	COO Hans [31]	COO gneSeqCOO [32]	DHIT/DZSig [10]	Genetic subtype
DB	DLBCL‐NOS	EZB	EZB	C3	GCB	GCB	DHIT/Dzsig‐pos	EZB/MYC+
DoHH2	HGBL‐DH‐*BCL2*	EZB	EZB	C3	GCB	GCB	DHIT/Dzsig‐pos	EZB/MYC+
Farage	DLBCL‐NOS	EZB	UNCL.	C3	GCB	GCB	DHIT/Dzsig‐neg	EZB
HBL‐1	DLBCL‐NOS	MCD	MCD	C5	Non‐GCB	ABC	—	MCD
HT	DLBCL‐NOS	ST2	ST2	C2^a^	GCB	GCB	DHIT/Dzsig‐neg	ST2
Karpas‐231	HGBL‐DH‐*BCL2*	EZB	EZB	C3	GCB	GCB	DHIT/Dzsig‐pos	EZB/MYC+
Karpas‐422	DLBCL‐NOS	EZB	EZB	C3	GCB	GCB	DHIT/Dzsig‐interm	EZB
MD901	HGBL‐DH‐*BCL2*	EZB	EZB	C3	GCB	GCB	DHIT/Dzsig‐pos	EZB/MYC+
NU‐DHL‐1	HGBL‐DH‐*BCL2*	EZB	EZB	C3	Non‐GCB	GCB	DHIT/Dzsig‐interm	EZB/MYC+
NU‐DUL‐1	DLBCL‐NOS	UNCL.	UNCL.	C3	GCB	UNCL.	DHIT/Dzsig‐pos	NC
OCI‐Ly1	HGBL‐DH‐*BCL2*	EZB	EZB	C3	GCB	GCB	DHIT/Dzsig‐pos	EZB/MYC+
OCI‐Ly10	DLBCL‐NOS	MCD	MCD	C5	Non‐GCB	ABC	—	MCD
OCI‐Ly19	HGBL‐DH‐*BCL2*	EZB	EZB	C3	GCB	GCB	DHIT/Dzsig‐pos	EZB/MYC+
OCI‐Ly3	DLBCL‐NOS	MCD	MCD	C5	Non‐GCB	ABC	—	MCD
OCI‐Ly7	HGBL‐DH‐*BCL2*	UNCL.	UNCL.	C1^b^	GCB	GCB	DHIT/Dzsig‐neg	NC
OCI‐Ly8	HGBL‐DH‐*BCL2*	EZB	EZB	C3	GCB	GCB	DHIT/Dzsig‐pos	EZB/MYC+
Pfeiffer	DLBCL‐NOS	EZB	EZB	C3	GCB	UNCL.	DHIT/Dzsig‐neg	EZB
RIVA	DLBCL‐NOS^#^	BN2	BN2	C1	Non‐GCB	ABC	—	BN2
RL	DLBCL‐NOS	EZB	EZB	C3	GCB	GCB	DHIT/Dzsig‐interm	EZB
SU‐DHL‐4	HGBL‐DH‐*BCL2*	EZB	EZB	C3	GCB	GCB	DHIT/Dzsig‐pos	EZB/MYC+
SU‐DHL‐5	DLBCL‐NOS	ST2	ST2	C4	GCB	GCB	DHIT/Dzsig‐neg	ST2
SU‐DHL‐6	HGBL‐DH‐*BCL2*	EZB	EZB	C3	GCB	GCB	DHIT/Dzsig‐pos	EZB/MYC+
SU‐DHL‐8	DLBCL‐NOS	ST2	ST2	C4	Non‐GCB	GCB	DHIT/Dzsig‐interm	ST2
SU‐DHL‐10	HGBL‐DH‐*BCL2*	EZB	EZB	C3	GCB	GCB	DHIT/Dzsig‐interm	EZB/MYC+
Toledo	HGBL‐DH‐*BCL2*	EZB	EZB	C3	GCB	GCB	DHIT/Dzsig‐pos	EZB/MYC+
U‐2932	DLBCL‐NOS	ST2	UNCL.	C2	GCB	UNCL.	DHIT/Dzsig‐neg	ST2
VAL	HGBL‐DH‐*BCL2*	EZB	EZB	C3	GCB	GCB	DHIT/Dzsig‐pos	EZB/MYC+
WSU‐DLCL2	HGBL‐DH‐*BCL2*	EZB	EZB	C3	GCB	GCB	DHIT/Dzsig‐interm	EZB/MYC+
WSU‐NHL	DLBCL‐NOS	N1	N1	C3	Non‐GCB	GCB	DHIT/Dzsig‐neg	N1

*Note:* Classifiers equivalences; C1 = BN2; C2 = A53; C3 = EZB; C4 = ST2; C5 = MCD; UNCL = Unclassified; #: HGBL‐DH‐*BCL6*.

^a^ C4 in reference [16].

^b^ C2 in reference [16].

Targeted DNA sequencing detected somatic mutations in all lines, with a mean of 7.5 mutated genes (range: 2‐19). Several genes exhibited multiple mutations per line, particularly *BCL2*, *MYC, SGK1* (associated with aberrant somatic hypermutation [aSHM]), *KMT2D* and *TP53.* The most frequently mutated genes were *TP53* (59%)*, CREBBP* (55%)*, BCL2* (52%), and *KMT2D* (48%), followed by *MYC* (41%), *EZH2* (34%), *GNA13* (31%), *FOXO1* (28%), *EP300* (24%), *CSMD3* (24%) and *H1‐4* (21%) (Figure 1B and Supporting Information S3: Table S4).

Comparison with external datasets (COSMIC, CCLE, MSK, Cell Model Passports) showed high concordance. 305 of 335 variants (91%) matched at least one external dataset. Only 9% (30/335) were unique to our analysis (Figure 1C and Supporting Information S3: Table S5). OCI‐Ly10, SU‐DHL‐5 and Farage showed the highest discrepancy rates (above 25%) (Figure 1C and Supporting Information S2: Figure S2A). Genes mutated in multiple lines with the greatest discordance were *BTG1*, *CSMD3*, *MYC*, and *H1‐4* (Figure 1C; Supporting Information S3: Table S5 and Supporting Information S2: Figure S2B).

The mutational data and *BCL2* and *BCL6* translocations were used to classify the DLBCL lines into genetic subtypes using the 2‐S [19], LymphGen [14], and DLBclass [16] classifiers. For 2‐S, we refined the *NOTCH1*/*NOTCH2* criteria to include only truncating PEST‐domain mutations. One line was classified as N1^2−S^ (WSU‐NHL), one as BN2^2−S^ (RIVA), three as MCD^2−S^ (OCI‐Ly10, HBL‐1 and OCI‐Ly3), 18 as EZB^2−S^ (DB, DoHH2, Farage, Karpas‐231, Karpas‐422, MD901, NU‐DHL‐1, OCI‐Ly1, OCI‐Ly8, OCI‐Ly19, Pfeiffer, RL, SU‐DHL‐4, SU‐DHL‐6, SU‐DHL‐10, Toledo, VAL, and WSU‐DLCL2), and four as ST2^2−S^ (U‐2932, SU‐DHL‐5, HT and SU‐DHL‐8). Of note, WSU‐NHL, classified as N1^2−S^, also had several mutations characteristic of the EZB^2−S^ genotype. NU‐DUL‐1 and OCI‐Ly7 remained unclassified (Table 1 and Figure 1B). The LymphGen classifier [14] yielded consistent classification with our mutational data, except for Farage (EZB^2−S^) and U‐2932 (ST2^2−S^), which were classified as “others” by LymphGen (Table 1 and Figure 1B). Therefore, the 2‐S algorithm and LymphGen achieved 93% agreement (27/29; Cohen's Kappa = 0.885), supporting robust genetic subtyping from complementary inputs. Similarly, we applied the DLBclass molecular classifier [16] using our mutational data (without CNV) and assigned the equivalent subtypes for most lines, observing 82.7% agreement between 2‐S and DLBclass (24/29; Cohen's Kappa = 0.686), with discrepancies in HT, U‐2932, OCI‐Ly7, and WSU‐NHL (Table 1 and Figure 1B, DLBCL Cell Line Explorer). However, in the original paper describing the DLBclass algorithm [16], which used mutational data from Whole‐Exome Sequencing (WES) and CNV data, HT was classified as C4/ST2, the same as the 2‐S algorithm, and OCI‐Ly7 was classified as C2/A53, increasing the concordance to 86.2% (25/29; Cohen's Kappa = 0.747). Thus, 2‐S assignments showed strong concordance with LymphGen and DLBclass classifiers.

### 3.2 Cell‐of‐Origin Classification of the DLBCL Cell Lines

**FIGURE 2:**
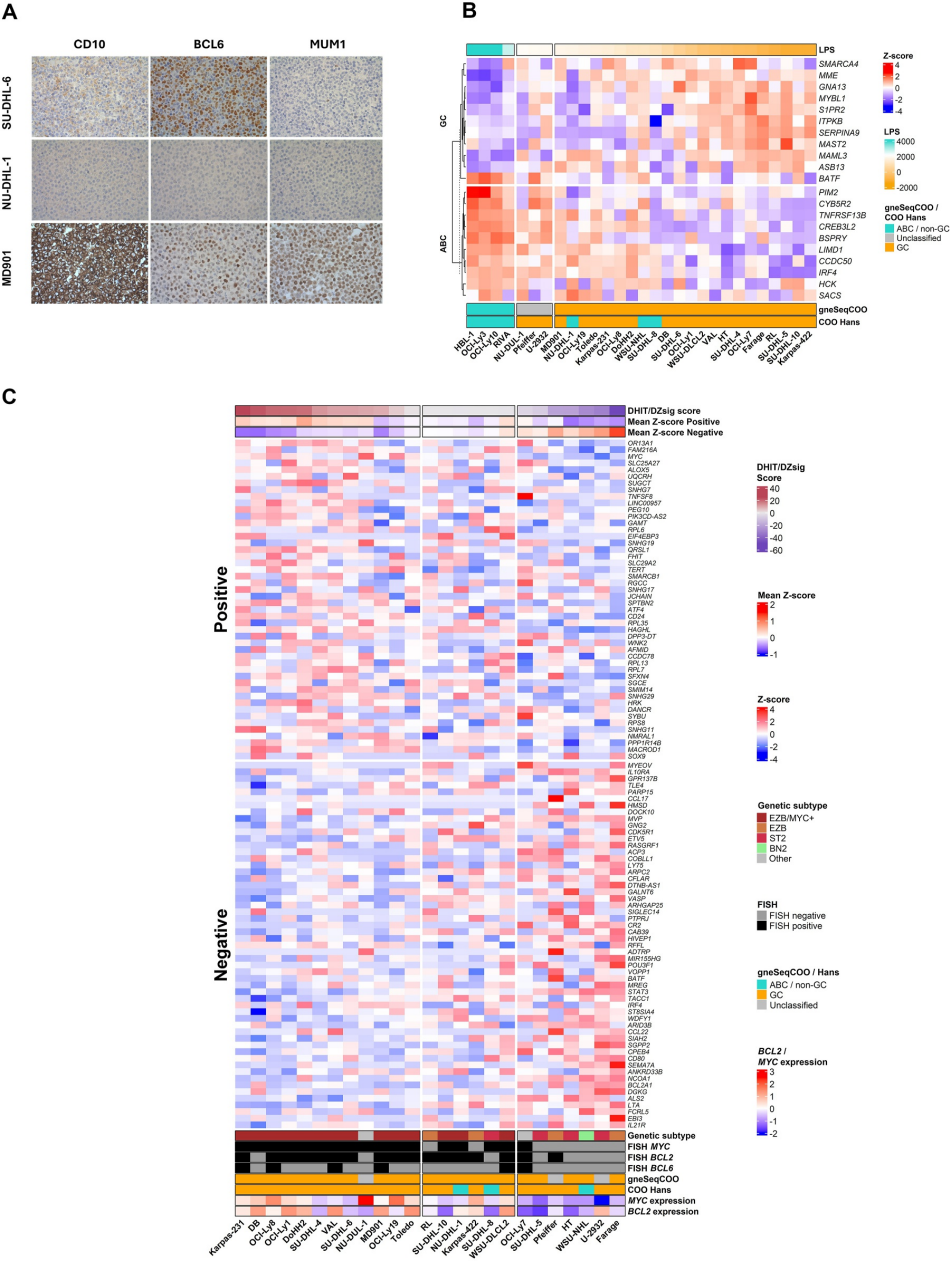
Identification of cell‐of‐origin and DHIT/DZ signature in the diffuse large B‐cell lymphoma‐derived cell lines. (A) Immunohistochemical analysis (IHC) of CD10, BCL6, and MUM1 protein expression in three selected diffuse large B‐cell lymphoma (DLBCL) cell lines: SU‐DHL‐6, classified as GCB by Hans' and gneSeqCOO; NU‐DHL‐1, classified as non‐GCB by the Hans' algorithm and GCB by gneSeqCOO; and MD901, classified as GCB by the Hans' algorithm and gneSeqCOO. Images were captured with a 40x objective. (B) Gene expression profiling of key genes in the gneSeqCOO signature was presented as a heatmap. LPS: Linear Predictor Score. (C) Transcriptomic DHIT/DZ signature analysis of the DLBCL‐derived cell lines. Gene expression profiling of 103 genes in the DHIT/DZ signature (DHIT/DZsig) is presented as a heatmap. The mean Z‐score of genes that are over‐ or under‐expressed is also shown. The figure displays the corresponding genetic subtype, the translocations of *MYC*, *BCL2*, and *BCL6* genes, and the cell‐of‐origin (COO) classifications determined by gneSeqCOO (activated B‐cells [ABC], germinal center B‐cells [GC] or unclassified) and Hans' algorithm (non‐GC or GC). mRNA levels of *MYC* and *BCL2* transcripts are also included.

We classified the 29 DLBCL lines based on COO using IHC and RNA‐seq data. Hans' algorithm [31] identified seven non‐GCB lines (HBL‐1, OCI‐Ly3, OCI‐Ly10, RIVA, NU‐DHL‐1, SU‐DHL‐8 and WSU‐NHL) (Table 1 and Supporting Information S3: Table S3, and Figure 2A) with all others classified as GCB. Notably, NU‐DHL‐1 was non‐GCB despite being HGBL‐DH‐*BCL2* (Table 1).

Using gneSeqCOO [32], 22 lines were GCB, largely concordant with IHC except for NU‐DHL‐1, SU‐DHL‐8, and WSU‐NHL (Supporting Information S3: Table S6). HBL‐1, OCI‐Ly3, OCI‐Ly10, and RIVA were ABC by gneSeqCOO and non‐GCB by IHC. NU‐DUL‐1, Pfeiffer, U‐2932 were GCB by IHC but unclassified by gneSeqCOO (Figure 2B, Table 1 and Supporting Information S3: Table S6, DLBCL Cell Line Explorer).

### 3.3 DHIT/DZ Signature and EZB Subclassification

Following Wright et al. [14], we applied the DHIT/DZsig [10, 11] to subdivide EZB lines into EZB/MYC‐ (no *MYC* translocation and DHIT/DZsig‐negative [neg]) and EZB/MYC+ (*MYC* translocation or DHIT/DZsig‐positive [pos]) subgroups.

We first classified GCB lines as DHIT/DZsig‐pos or ‐neg. Twelve lines were DHIT/DZsig‐pos, and all carried *MYC* translocations (Karpas‐231, DB, OCI‐Ly8, OCI‐Ly1, DoHH2, SU‐DHL‐4, VAL, SU‐DHL‐6, NU‐DUL‐1, MD901, OCI‐Ly19 and Toledo). Seven were DHIT/DZsig‐neg (Farage, SU‐DHL‐5, HT, U‐2932, Pfeiffer, WSU‐NHL and OCI‐Ly7), including two EZB, three ST2, the N1 and OCI‐Ly7 (unclassified) lines. Six lines exhibited intermediate signature (Karpas‐422, SU‐DHL‐10, RL, WSU‐DLCL2, NU‐DHL‐1, and SU‐DHL8) (Supporting Information S3: Table S6 and Figure 2C). Notably, DB and NU‐DUL‐1 were DHIT/DZsig‐pos despite lacking DH status. Conversely, three DH lines showed an intermediate DHIT/DZsig: NU‐DHL‐1, SU‐DHL‐10 and WSU‐DLCL2, emphasizing the complex genetic landscape in this lymphoma subtype (Table 1).

Among eighteen EZB‐classified lines, 14 were EZB/MYC+ (all with *MYC* translocation, 11 DHIT/DZsig‐pos): DB, DoHH2, Karpas‐231, MD901, NU‐DHL‐1, OCI‐Ly1, OCI‐Ly19, OCI‐Ly8, SU‐DHL‐10, SU‐DHL‐4, SU‐DHL‐6, Toledo, VAL, and WSU‐DLCL2. Four lines were EZB/MYC‐ (Farage, Karpas‐422, Pfeiffer, RL) (Figure 2C and Table 1, DLBCL Cell Line Explorer).

### 3.4 Transcriptomic Characterization of the DLBCL Cell Lines

To investigate transcriptional heterogeneity underlying DLBCL, we analyzed RNA‐seq data using differential expression (DE), GSEA, and ssGSEA. PCA was first performed to identify major expression patterns. MCD lines formed a tight and distinct cluster, reflecting a characteristic MCD transcriptional program (Supporting Information S2: Figure S3A). WSU‐NHL, genetically classified as N1 but carrying EZB‐genetic alterations, displayed an intermediate profile between MCD and the remaining lines. OCI‐Ly7, unclassified by 2‐S/LymphGen, was also segregated. Most EZB, ST2 and BN2 lines were grouped. When PCA was colored by gneSeqCOO, two large groups corresponding to GCB‐ and ABC‐DLBCL subtypes emerged, except for RIVA (Supporting Information S2: Figure S3B). A refined PCA excluding the five most divergent lines revealed more apparent clustering according to DHIT/DZsig status rather than diagnostic category (DLBCL‐NOS vs. HGBL‐DH‐BCL2) (Supporting Information S2: Figures S3C and S3D).

To characterize pathway‐level differences, ssGSEA was performed using the curated lymphoma gene‐set collection [33, 34] and Wright et al. [14] gene sets database (Supporting Information S3: Table S7). Unsupervised clustering of ssGSEA scores (Supporting Information S2: Figure S4 and Supporting Information S3: Table S8) revealed a distinct branch comprising the MCD lines with WSU‐NHL and OCI‐Ly7, consistent with the PCA (Supporting Information S2: Figure S3A).

**FIGURE 3:**
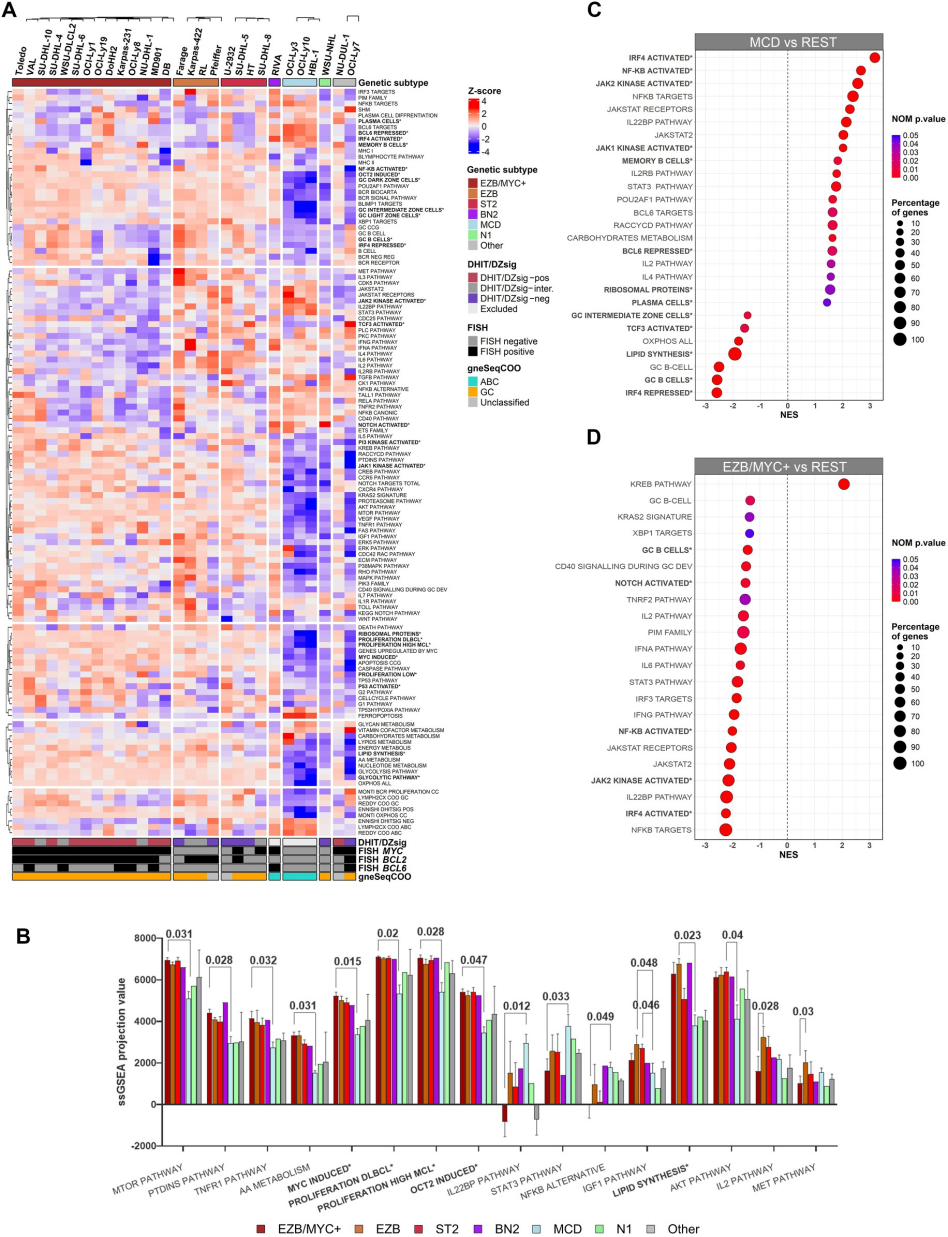
Pathway enrichment analysis in diffuse large B‐cell lymphoma‐derived cell lines based on the genetic subtypes. (A) Heatmap of the single‐sample gene set enrichment analysis (ssGSEA) analyzing key pathways associated with lymphoma pathogenesis, categorized by diffuse large B‐cell lymphoma (DLBCL) genetic subtype. The figure also displays the DHIT/DZ signature (DHIT/DZsig) score, as well as *MYC, BCL2,* and *BCL6* translocations, and the cell‐of‐origin (COO) classification (activated B‐cells [ABC], germinal center B‐cells [GC] or unclassified). (B) Bar plot showing differentially enriched pathways. The height of each bar indicates the mean ssGSEA projection value ± standard deviation, representing the level of pathway enrichment. Statistical significance was assessed using the Kruskal‐Wallis's test and Dunn's post hoc test for pairwise comparisons. A p‐adj. value < 0.05 was considered statistically significant. (C) The normalized enrichment score (NES) of indicated gene sets was used to compare gene expression in the MCD subtype vs. the other DLBCL cell lines, and (D) EZB/MYC+ subtype versus the other DLBCL cell lines. A positive NES indicates gene set enrichment at the top of the ranked list, while a negative NES shows enrichment at the bottom. Gene sets from Wright et al. [14] are indicated in bold and with an asterisk (*).

Heatmap visualization (Figure 3A), and Kruskal–Wallis and Dunn tests identified gene sets that differed significantly between genetic subtypes (Supporting Information S3: Table S9 and Figure 3B). MCD showed the most significant differences, particularly compared with the EZB/MYC+ subtype, with notable alterations in key signaling pathways. These included mTOR, phosphoinositide (PTDINS) and their downstream targets, TNFR1 pathways and amino acid (AA) metabolism, which were enriched in the EZB/MYC+ subtype, together with some signatures of malignant attributes, such as MYC activity (MYC induced) and proliferation (proliferation DLBCL and HIGH MCL), and high expression of OCT2 and its target genes (OCT induced). Conversely, the MCD lines showed greater activation of the IL22BP, STAT3, and NF‐κB pathways than the EZB/MYC+ subtype. Additional differences included enrichment of IGF1 signaling and lipid synthesis in EZB versus MCD subtypes, and higher AKT and IGF1 pathway activity in ST2 versus MCD subtypes. Within EZB, IL2 and MET signaling were higher in EZB than in EZB/MYC+ subtype (Figure 3A and 3B).

Pairwise GSEAs further supported these observations and provided additional insights. MCD showed downregulation of GC‐related signatures (e.g., IRF4‐repressed, OxPhos), and strong upregulation of NF‐κB, JAK/STAT, IL22BP, TNF, and IRF4‐activated programs (Figure 3C, Supporting Information S2: Figure S5, Supporting Information S3: Tables S10 and S11) consistent with patient MCD signatures [14].

Meanwhile, EZB/MYC+ was enriched for the KREB pathway, E2F and MYC targets and mitotic spindle and other cell‐cycle pathways, while showing reduced JAK/STAT, NFκB and IL‐related pathways relative to EZB and ST2 (Figure 3D and Supporting Information S2: Figure S6). Interestingly, EZB/MYC+ exhibited downregulation of proteasome activity and IL22BP compared to ST2 (Supporting Information S2: Figure S6B). These features align with EZB/MYC+ patient samples, including reduced NF‐κB/JAK–STAT pathway, NOTCH and IRF4 activity [14].

EZB lines displayed transcriptional profiles that mirrored those of EZB lymphomas, including elevated GC B‐cell signatures and reduced memory B‐cell and IRF4‐activated signatures (Supporting Information S2: Figure S7A‐D). Similarly, ST2 lines showed activation of DNA repair mechanisms (Supporting Information S2: Figure S7E). EZB upregulated IRF4‐repressed target genes, reinforcing the consistency between cell line models and primary tumor biology in this subtype (Supporting Information S3: Table S10 and Supporting Information S2: Figure S7B). Full GSEA profiles for all comparisons are included in the supplemental tables (Supporting Information S3: Tables S10 and S11 and Supporting Information S2: Figures S5‐S7).

**FIGURE 4:**
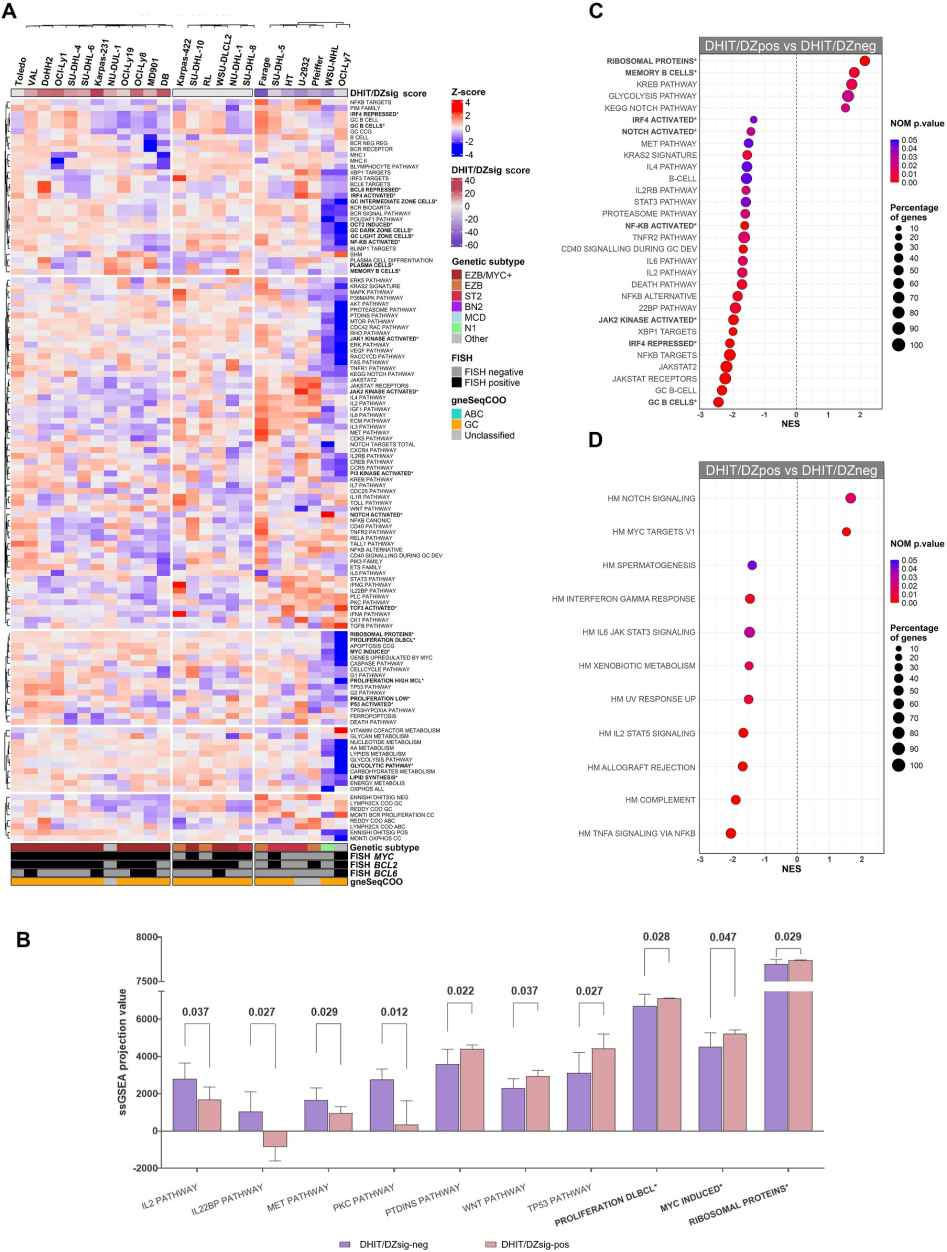
Pathway enrichment analysis in diffuse large B‐cell lymphoma‐derived cell lines based on DHIT/DZ signature. (A) Heatmap of single‐sample gene set enrichment analysis (ssGSEA) categorized by DHIT/DZ signature (DHIT/DZsig). The figure also includes the genetic subtypes, along with the *MYC*, *BCL2*, and *BCL6* translocations, as well as the cell‐of‐origin (COO) classification by gnSeq COO (germinal center [GC] or unclassified). (B) Bar plot showing differentially enriched pathways. The height of each bar indicates the mean ssGSEA projection value ± standard deviation, reflecting the level of pathway enrichment in DHIT/DZsig‐positive and DHIT/DZsig‐negative cell lines. Statistical significance was assessed using the Kruskal‐Wallis's test and Dunn's post hoc test for pairwise comparisons. A p.adj value < 0.05 was deemed statistically significant. (C) Normalized enrichment score (NES) of significant gene sets after GSEA with the combined curated Lymphoma/Wright database in DHIT/DZsig‐positive (DHIT/DZ‐pos) versus DHIT/DZsig‐negative (DHIT/DZ‐neg) cell lines, and (D) with Hallmark signatures from the Broad Institute. A positive NES indicates enrichment of the gene set, while a negative NES suggests downregulation. Gene sets from Wright et al. [14] are indicated in bold and with an asterisk (*).

We next examined transcriptional differences between DHIT/DZsig‐pos and DHIT/DZsig‐neg cell lines by ssGSEA visualization (Supporting Information S3: Table S8) as a heatmap (Figure 4A) and statistical testing (Supporting Information S3: Table S9). DHIT/DZsig‐pos lines showed lower expression of IL2, IL22BP, MET, and PKC pathways and higher activity in PTDINS, WNT, TP53, proliferation‐related and MYC‐associated signatures, and ribosomal proteins (Figure 4B).

GSEA further demonstrated that DHIT/DZsig‐pos lines were enriched in ribosomal proteins, MYC targets, Krebs‐cycle pathways, and reduced expression of JAK/STAT, GC B cells, NF‐κB, IRF4, TNF, and IL‐related signatures (Figure 4C and 4D, Supporting Information S3: Tables S10 and S11). These findings are consistent with DHIT/DZsig‐pos patient samples [10], which similarly show upregulated MYC programs and reduced apoptosis, TNFα, IL‐2/STAT5 signaling, and interferon‐related pathways.

### 3.5 Regulon Analysis

**FIGURE 5:**
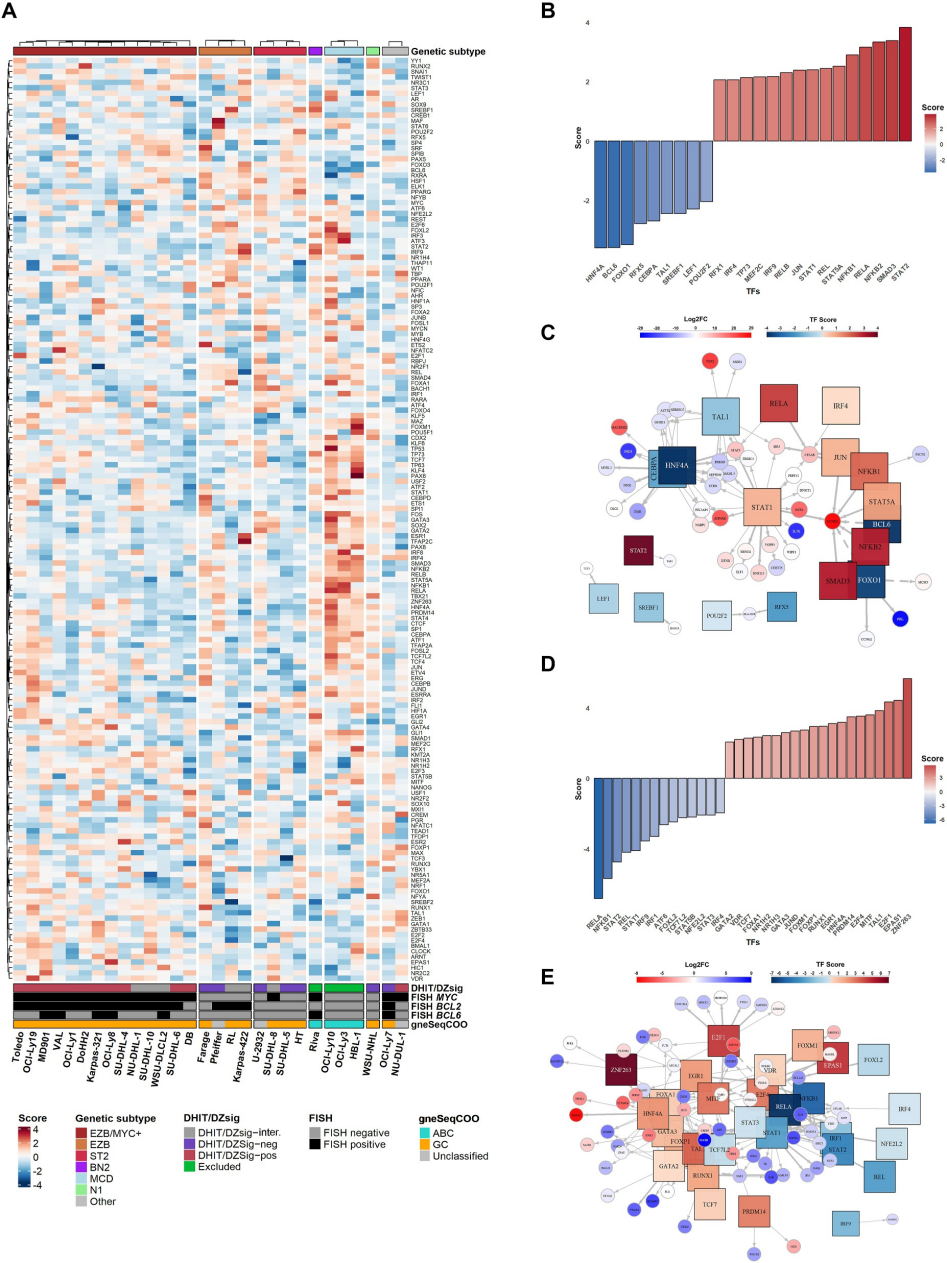
Regulon analysis profiles in diffuse large B‐cell lymphoma‐derived cell lines based on genetic subtype. (A) A regulon activity analysis was performed on significantly active transcription factors (TFs) to identify key TFs and their main target genes, as well as to explore how TFs influence gene expression across different genetic subtypes. The results are presented as a heatmap. The figure also displays the DHIT/DZ signature (DHIT/DZsig) classification, the translocations of *MYC*, *BCL2* and *BCL6*, genes and the cell‐of‐origin (COO) classification by gneSeqCOO (activated B‐cells [ABC], germinal center B‐cells [GC] or unclassified). (B) Bar plot showing the TF inferred activity score for MCD compared to other cell lines. (C) Network diagrams illustrating differentially activated TFs and their targets in MCD cell lines vs. others. (D) Bar plot displaying the TF inferred activity score for EZB/MYC+ compared to other cell lines. (E) Network diagrams comparing differentially activated TFs and their targets between EZB/MYC+ and the remaining cell lines.

We performed regulatory network analysis with DoRothEA [35] to identify active TFs and their target genes across genetic subtypes (Supporting Information S3: Table S12). MCD lines showed a clearly distinct regulon profile (Figure 5A), with strong upregulation of NF‐κB‐related TFs (NFKB1/2, REL, RELA/B) and STAT TFs (STAT1/2/5A) (Figure 5B), which promote expression of genes, such as *CCND2* (Figure 5C), contributing to heightened proliferation typical of ABC‐type biology [36, 37]. Several TFs were downregulated, including BCL6, FOXO1, HNF4A, and POU2F2; reduced BCL6 and FOXO1 activity may further enhance CCND2 expression. Detailed results for pairwise comparisons are provided in the Supplemental Data (Supporting Information S2: Figure S8).

In EZB/MYC+ lines, TFs related to increased proliferation and cell cycle regulation (E2F1 and E2F4) were upregulated as previously reported in DHIT/DZsig‐pos patients [10], but also new TFs such as ZNF263, EPAS1, MITF, and TAL1, not previously found related to EZB/MYC+ lymphomas (Figure 5A, 5D and 5E). Downregulated TFs included RELA, REL, NFKB1, IRF9, STAT1, and STAT2, which regulate immune and inflammatory genes (e.g., *SOCS3*, *IL6R*, *IL10*, *CD83*, *BIRC3*) (Figure 5E). Differences between EZB/MYC^+^ and EZB mainly reflected activation of proliferation‐related TFs versus NF‐κB/STAT TFs, consistent with GSEA findings (Supporting Information S2: Figure S9A). Detailed results for pairwise comparisons are provided in the Supplemental Data (Supporting Information S2: Figure S9 and Supporting Information S3: Table S12).

## 4 Discussion

Few studies have comprehensively characterized B‐cell lymphoma cell lines, particularly DLBCL‐NOS and HGBL‐DH‐*BCL2*. Our work bridges this gap by integrating genetic, transcriptomic, and immunophenotypic analyses of 29 DLBCL lines, providing a valuable reference for functional studies of aggressive B‐cell lymphomas (DLBCL Cell Line Explorer).

We classified 14 lines as HGBL‐DH‐*BCL2*, 6 of which also carried a *BCL6* rearrangement. Several discrepancies emerged when comparing *MYC*, *BCL2* and *BCL6* translocations to previous reports. We could not detect *BCL2* translocation in DB and SU‐DHL‐5, which were previously classified as HGBL‐DH‐*BCL2* [26], whereas OCI‐Ly3 exhibited only gene amplifications [38]. These differences likely reflect methodological variations, FISH sensitivity, probe types, or locations [39], underscoring the need for standardized characterization, as cryptic or complex rearrangements may remain undetected.

*TP53* mutations were the most frequent (59%), markedly higher than the 10%–30% reported in primary DLBCL samples [14, 15, 16, 19, 23]. This fact may stem from the high‐grade nature of our panel or the tendency of long‐term cultures to acquire *TP53* mutations [21]. Other recurrent mutations showed high concordance with previous studies, with 90% of mutations validated in external datasets [13, 15, 17, 19, 23, 40]. Discrepancies primarily involved genes prone to aSHM (*MYC* or *BTG1*) or long, mutation‐susceptible genes (*CSMD3*). High discrepancies in *H1‐4* likely reflect challenges in annotating histone‐coding genes.

Genetic subtyping showed strong concordance between the 2‐S and LymphGen classifiers, with 27 out of 29 lines assigned the same subtype. Notably, Farage has been previously described as plasmablastic B‐cell lymphoma [25], supported by its distinct GEP. DLBclass largely agreed with these assignments, although discrepancies arose from differences in the required input features. WSU‐NHL was classified as C3/EZB by DLBclass, but the tool lacks an N1 equivalent, a clinically relevant subtype we believe should be preserved [14, 17, 19] (Table 1). These results further support the accuracy of 2‐S, which relies on FISH‐based *BCL2* and *BCL6* rearrangements and 26 genes, compared to the 163 features, including CNVs, required by DLBclass [16]. The high concordance among classifiers confirms that 2‐S is a robust and reliable tool for DLBCL genetic classification, with the added advantage of being simpler to apply.

COO classification by IHC and GEP revealed inconsistencies, as previously reported [4]. NU‐DHL‐1, WSU‐NHL and SU‐DHL‐8 were GCB by gneSeqCOO but non‐GCB by IHC. While most lines matched prior reports [27, 41, 42, 43, 44, 45], two unclassified lines by gneSeqCOO (NU‐DUL‐1, U‐2932) were GCB by IHC, despite prior identification as ABC [27, 42].

A key observation is that several DLBCL‐NOS lines exhibit a high‐grade biology profile despite lacking *MYC*/*BCL2* translocations. Our DHIT/DZsig classifier identified several DLBCL‐NOS lines as DHIT/DZsig‐pos, while some HGBL‐DH‐*BCL2* lines were DHIT/DZsig‐intermediate, suggesting that GEP may capture high‐grade biology beyond translocation detection. Although our DHIT/DZsig results rely on cohort‐based comparisons rather than independent assessment [10, 46], the expression patterns we observed (MYC target upregulation with reduced NF‐κB, JAK/STAT, TNF, and interferon signaling) mirror findings from primary tumors, supporting the biological relevance of this classifier [10].

Transcriptomic and regulon analyses validated the molecular subtypes, consistent with findings in primary DLBCL [13, 14]. MCD and N1 lines exhibited distinct profiles, including downregulation of mTOR, TNF signaling and amino acid metabolism pathways, but activation of JAK/STAT, NF‐kB and IRF4 signaling, consistent with poor clinical outcomes [47], and IL22BP, whose downregulation has been demonstrated to depend on MyD88 signaling in murine models [48]. EZB/MYC+ lines showed significant enrichment in metabolic, MYC‐driven, and cell‐cycle pathways, with reduced inflammatory and stress‐response signaling, indicating dependence on proliferative programs.

## 5 Conclusion

Our study provides a detailed molecular and phenotypic analysis of DLBCL cell lines by combining FISH, targeted sequencing, and transcriptomic data to establish a reliable framework for selecting experimental models. The strong agreement among the 2‐S, LymphGen, and DLBclass classifiers supports their robustness, with the 2‐S algorithm standing out as a practical and efficient option for genetic subtyping. We also show that FISH has limitations in identifying HGBL‐DH‐*BCL2* cases and that DHIT/DZsig profiling helps reveal HGBL‐like features. Overall, these findings enhance the translational value of DLBCL cell lines and provide clinically relevant insights to improve risk stratification and guide precision therapy.

## Author Contributions

M.P.‐A, L.P., A.O.‐M. and M.S.‐B: conceptualization. M.P.‐A, L.P., S.G., S.S.‐O., P.M., B.L.M.‐L., R.J., A.O.‐M. and M.S.‐B.: methodology. M.P.‐A, L.P., I.F.‐M., R.M.‐V. A.O.‐M. and M.S.‐B: data curation. L.P., S.G., S.S.‐O., I.F.‐M., R.M‐V, P.M., B.L.M.‐L., R.J., N. Y.‐C., and B.H.: investigation: M.P.‐A, L.P.: validation. M.P.‐A, L.P., S.S.‐O., I.F.‐M., R.M‐V, P.M., B.L.M.‐L., R.J., A.O.‐M. and M.S.‐B.: formal analysis. A.O.‐M. and M.S.‐B: supervision. A.O.‐M. and M.S.‐B: funding acquisition. M.P.‐A, L.P., I.F.‐M., R.M‐V, P.M., A.O.‐M. and M.S.‐B: visualization. A.O.‐M. and M.S.‐B: project administration. G.R., J.A.M.‐C. A.O.‐M. and M.S.‐B: resources. M.P.‐A., S.S.‐O., B.L.M.‐L., R.J., A.O.‐M. and M.S.‐B.: writing – original draft. All authors: writing – review and editing.

## Funding

The work at IDIPHISA has been funded by the Instituto de Salud Carlos III (ISCIII) and co‐funded by the European Union (grants PI20/00591, PI23/01587) under the State Plan for Scientific, Technical and Innovation Research within the Recovery, Transformation and Resilience Plan; the Regional Ministry of Education and Research of the Regional Government of Comunidad de Madrid (CM) (grant B2017/BMD‐3778); Spanish State Research Agency, co‐funded by the European Regional Development Fund (CPP2023/01016) and the Fundación de Investigación Biomédica H.U. Puerta de Hierro‐Majadahonda (FIBHUPHM). The work at CBM CSIC‐UAM was supported by a Generación de Conocimiento Project under the Program of the Spanish Ministry of Science, Innovation and Universities/Spanish State Research Agency/European Regional Development Fund (PID2021‐124601OB‐I00/AEI/10.13039/501100011033/FEDER, UE; PRE2022‐101597; PRE2022‐101598; RYC2019‐027280‐I/AEI/10.13039/501100011033); Fundacion Cientifica Asociacion Española Crontra El Cancer (FCAECC; LABAE235101ORTE); FERO Foundation (BFERO2021.05); BBVA Foundation (LEO22‐2‐2003); ASEICA+queuntrail; Consejo Superior de Investigaciones Cientificas (20212AT020). M.P.‐A. was supported by Plan de Empleo Juvenil from CM (PEJ‐2023‐AI/SAL‐GL‐28806) and is recipient of a *Ayuda para la contratación de personal investigador predoctoral* contract from CM (PIPF‐2023/SAL‐GL‐29762); L.P. was a recipient of an iPFIS predoctoral fellowship (IFI18/0004, ISCIII‐MICIU AES‐FEDER, Plan Estatal I + D + I 2014‐2020). S.S.‐O. is a recipient of a *Ayuda de Contratos Predoctorales para la formación de Doctores* from MICIU/AEI (PRE2022‐101597). I.F.M. was supported by B2017/BMD‐3778 (CM) and FIB HUPHM. B.L.M.‐L. was supported by *Programa Investigo* funded by the European Union—Next Generation EU from CM and is a recipient of a *Ayuda de Contratos Predoctorales para la formación de Doctores* from MICIU/AEI (PRE2022‐101598). R.J. was supported by a Consejo Superior de Investigaciones Cientifícas (20212AT020) grant. N.Y.‐C. was supported by the *Asociación Española Contra el Cáncer*. B.H. was supported by the *Plan de Empleo Juvenil* from CM (PEJ‐2020‐TL/BMD‐19530). A.O.‐M. is a recipient of a Ramon y Cajal Award from MICIU/AEI (RYC2019‐027280‐I/AEI/10.13039/501100011033). J.A.M.‐C. was supported by grants from the Spanish Ministry of Health ISCIII‐FIS PI22/00983, CIBERONC‐CB16/12/00489, and Fundacion Arnal Planelles. The Centro de Biologia Molecular Severo Ochoa is the recipient of a “Severo Ochoa Excellence” grant (CEX2021‐001154‐S) funded by MICIU/AEI (10.13039/501100011033) and receives institutional support from the Ramon Areces Foundation.

## Ethics Statement

The authors have nothing to report.

## Consent

The authors have nothing to report.

## Conflicts of Interest

J.A.M.‐C. obtained research funding from Roche‐Genentech, BMS‐Celgene, Janssen, Priothera, Palleon, AstraZeneca and K36 Therapeutics, and is the founder and holds stock options in MIMO Biosciences. The remaining authors declare no competing financial interests.

## Permission to Reproduce Material From Other Sources

The authors have nothing to report.

## Supporting information


Supporting Information S1

Supporting Information S2

Supporting Information S3

## Data Availability

Sequence data supporting the findings of this study have been deposited in the Sequence Read Archive (SRA), under the accession number PRJNA1249247. The scripts used for data processing and downstream analyses are now available through the Lymphoma Research Group GitHub repository (https://github.com/Lymphoma‐IDIPHISA/). In addition, an interactive web resource, the DLBCL Cell Line Explorer Shiny app (https://lymphomaresearchgroup‐idiphisa.shinyapps.io/LymphomaCellLines/), is publicly available to visualize and explore the molecular data generated in this study. All other data supporting the findings of this study are available at Supporting Information S3: Tables and from the corresponding authors upon request.
