# Supplementary material for: Decoding the Heterogeneity of Diffuse Large B‐Cell Lymphomas: A Comprehensive Genetic, Transcriptomic, and Phenotypic Profiling of B‐Cell Lymphoma Cell Lines
Source: Hematol Oncol. 2026 Aug 5;44(5):e70228. doi: 10.1002/hon.70228 (PMC13440701; doi:10.1002/hon.70228)
Supplement: Supplementary file 1 [file HON-44-e70228-s002.pdf]

Supplemental Data for

Decoding the Heterogeneity of Diffuse Large B-Cell Lymphomas: A Comprehensive Genetic, Transcriptomic, and Phenotypic Profiling of B-Cell Lymphoma Cell Lines

Marina Pérez-Aguilera[1*], Lucía Pedrosa[1*], Sagrario Gómez[1], Sara Salazar-Ortego[2], Ismael Fernández-Miranda[1], Rafael Muñoz-Viana[3], Paloma Martín-Acosta[4], Beatriz L. Martín-Lunas[2], Rebeca Jimeno[2], Natalia Yanguas-Casás[1,5], Beatriz Horcajo[1], Giovana Roncador[6], José A. Martínez-Climent[7], Ana Ortega-Molina[2#], and Margarita Sánchez-Beato[1#].

[1]Lymphoma Research Group, Medical Oncology Department, Hospital Universitario Puerta de Hierro-Majadahonda, IDIPHISA, Madrid, Spain

[2]Metabolism in Cancer and Aging Research Group; Immune System Development and Function Department, Severo Ochoa Centre for Molecular Biology, CSIC-UAM, Madrid, Spain

[3]Bioinformatics Unit, Hospital Universitario Puerta de Hierro-Majadahonda, IDIPHISA, Madrid, Spain

[4]Cancer Molecular Pathology Group, Pathology Department, Hospital Puerta de Hierro-Majadahonda, IDIPHISA, Madrid, Spain

[5]Centro de Investigación Biomédica en Red de Fragilidad y Envejecimiento Saludable (CIBERFES), Madrid, Spain.

[6]Monoclonal Antibodies Core Unit Biotechnology Program Spanish National Cancer Research Centre (CNIO), Madrid, Spain.

[7]Department of Haematology, Center for Applied Medical Research, Cancer Center Clinica Universidad de Navarra, IDISNA, CIBERONC, Pamplona, Spain

SUPPLEMENTAL MATERIALS & METHODS

Cell lines and cell culture

Cell line authentication was performed by short tandem repeat (STR) profiling at the *Instituto de Investigaciones Biomédicas "Alberto Sols"* or at the *Centro Nacional de Investigaciones Oncológicas* (CNIO). Mycoplasma testing was performed regularly using the Mycoplasma Gel Detection Kit (Biotools B&M Labs, S.A., Madrid, Spain) or the PlasmoTest Mycoplasma Detection Kit (rep-pt1, InvivoGen). Cell culture conditions are listed in **Table S1**.

Tissue microarray, immunohistochemistry and fluorescence *in situ* hybridization

Formalin-fixed and paraffin-embedded cell line (FFPEcl) pellets were prepared, and two 1-mm cores per FFPEcl block were included in a tissue microarray (TMA) using a tissue arrayer (Beecher Instruments Inc). TMA sections were used for COO classification by immunohistochemistry (IHC) and for the detection of *MYC*, *BCL2* and *BCL6* fusions by fluorescence in situ hybridization (FISH).

The TMA sections were stained with monoclonal mouse antibodies from Agilent-Dako (Santa Clara, CA., USA): CD10 (clone DAK-CD10), BCL6 (PG-B6p), and MUM1 (MUM1p). The staining procedures were performed on the DAKO Omnis Platform (Agilent Technologies; Santa Clara, CA, USA) according to the routine protocol at the Pathology Department of H.U. Puerta de Hierro-Majadahonda. Hans' algorithm was used to establish the COO based on IHC [30]. Dual-color break-apart probes (ZytoVision GmbH, Bremerhaven, Germany) were used to analyze *BCL2* (18q21 region) (Z-2192-50), *BCL6* (3q27) (Z-2177-50), and C-*MYC* (8q24) (Z-2090-50; chr8:127,888,765-130,930,673) rearrangements by FISH, according to the manufacturer's instructions. Additional FISH analyses were conducted in DB, RL, SU-DHL4, and WSU-NHL fresh cell smears prepared immediately after sample collection using Vysis LSI IGH/BCL2 Dual Color,

Dual Fusion Translocation Probe (# 05J71-001) (Abbot, USA) and Vysis LSI MYC Dual Color Break Apart Rearrangement Probe (# 05J91-001; chr8:127,342,188-129,663,861) (Abbott, USA).

Bioinformatic analyses

DNA-sequencing

The targeted regions (according to the Human Assembly GRCh38/hg38) were captured using a Twist Target Enrichment with Mechanical Fragmentation kit, following the manufacturer's instructions (Twist Bioscience; San Francisco, CA., USA). Paired-end sequencing was performed on the MiSeq sequencer (Illumina, San Diego, CA., USA).

Raw reads were processed using the nf-core/sarek v3.4.3 pipeline [1,2] with Nextflow v24.04.4 with default options [3]. Raw reads were processed with fastp 0.23.4 [4], quality was assessed with fasqC v0.12.1 (RRID: SCR_014583) and then mapped to the human reference genome GRCh38 using Burrows-Wheeler Aligner v0.7.18 (BWA) [5] and processed with MarkDuplicates, BaseRecalibrator and ApplyBQSR from Genomic Analysis Toolkit v4.5 (GATK). Somatic variant calling was performed using Mutect2 [6] from GATK to detect SNVs and indels. Other tools included in the analysis were samtools v1.19.2 (RRID: SCR_002105), mosdepth v0.3.8 [7], vcftools v0.1.16 [8] and bcftools v1.2 [9]. Annotation was performed using Annovar v2024-08-22 [43]. All identified variants were visualized using Integrative Genomics Viewer (IGV) v2.18.2 (Broad Institute and UC San Diego). Data has been deposited in the Sequence Read Archive (SRA) (PRJNA1249247). After annotation, the variants were subjected to additional, more stringent, and quality- and relevance-based filtering applying the following criteria: depth coverage ≥ 50; depth of variant-supporting bases ≥ 5; localisation (exonic, and splice site); variant effect (non-synonymous); variant allele frequency

≥ 10% and not listed as a single nucleotide polymorphism, or listed but with a MAF < 0.01% (The Exome Aggregation Consortium, 1000 Genomes Project of the International Genome Sample Resource, Single Nucleotide Polymorphism Database (dbSNP) v151 of the National Center for Biotechnology Information.

The Oncoplot visualization was created using custom R scripts (R Project for Statistical Computing, v4.4.1, RRID: SCR_000432) with ComplexHeatmap v2.22.0 (RRID: SCR_017270).

The single nucleotide variations (SNVs) found were compared to the Catalogue Of Somatic Mutations In Cancer (COSMIC) version 101 [11], Lymphoma Cell Lines from MSK [12], the Cancer Cell Line Encyclopedia (CCLE) database [13] available from cBioPortal (https://www.cbioportal.org/), the Cell Model Passports database (https://cellmodelpassports.sanger.ac.uk/), data published by Morin *et al*. [14] and Tarantelli *et al*. [15]. A match was considered valid when the identified variant matched in at least one of the databases.

The genetic subtype was assigned using the 2-S© algorithm (https://github.com/Lymphoma-IDIPHISA/Two-step-classifier)[16], the LymphGen 2.0 classifier (https://llmpp.nih.gov/lymphgen/index.php) [17] and the new DLBclass classifier https://github.com/getzlab/DLBclass-tool) [18].

RNA-sequencing

For the 3' mRNA-Seq data analysis, ~ 10 million reads were generated from each library. FASTQ files were uploaded into the Lexogen Data Analysis Portal for primary quality control (QC) and transcript quantification. Reads were aligned to the human reference genome (GRCh38.p14

primary assembly) using HISAT2 v2.7.11b, annotated with Ensembl GRCh38 v113, and quantified with HTSeq v2.0.3.

Samples were processed across five technical batches (batch 1–5), defined by RNA extraction, library preparation and sequencing runs. Batch 1 included DB, DOHH2, SU-DHL-10 and WSU-DLBCL2; batch 2 included TOLEDO, SU-DHL-8 and OCI-LY7; batch 3 included HBL1, HT, OCI-LY3, OCI-LY10, OCI-LY19, RL, SU-DHL-4, SU-DHL-6, U2932, and WSU-NHL; batch 4 included FARAGE, KARPAS-321, MD901, NU-DUL-1, NU-DHL1, PFEIFFER, OCI-LY1, OCI-LY8, RIVA, SU-DHL-5 and VAL; and batch 5 included KARPAS-422. Most batches included a heterogeneous mix of genetic subtypes, thereby minimizing potential confounding between batch and biological variables. The only exceptions were the N1 subtype, represented by a single cell line (WSU-NHL), and Karpas-422, processed in a single batch.

Batch correction was performed on the count matrix using ComBat-seq from the sva package v3.52.0 (RRID:SCR_012836), correcting for technical batch effects while preserving genetic subtype-associated variation. To assess the effectiveness of batch correction, PCA analyses using ggfortify_v0.4.17 (RRID:SCR_024888) were conducted before and after correction, confirming a reduction in batch-associated variation while preserving biologically meaningful clustering according to genetic subtype. These batch-corrected counts were used for COO classification with the gneSeqCOO pipeline, which applies its own internal normalization procedure.

Batch-corrected counts were filtered (>10 counts in at least 3 samples (10%)) for downstream normalization with DESeq2 v1.44 (RRID:SCR_015687), including both library size normalization and variance-stabilizing transformation (VST).

The different expression matrices were used as follows:

- Raw reads, batch-corrected and filtered reads, were used for differential expression analyses between genetic subtypes, which were performed using DESeq2 with the design formula ~ batch + subtype. The differential TF activity was calculated using the stat statistic and decoupleR.
- VST-transformed batch-corrected counts were used for principal component analysis (PCA clustering, Transcription Factor (TF) activity scores using DoRothEA/decoupleR, and DHIT/DZsig classification. PCA was performed using the plotPCA function from DESeq2 and restricted to protein-coding genes.
- DESeq2 library-size normalized batch-corrected counts were used for pathway enrichment analyses (GSEA and ssGSEA).

A schematic overview of the RNA-seq analytical workflow, including the expression matrix used at each analytical step, is provided in Figure S1.

Cell-of-origin and DHIT/DZ signature classifications

We used gneSeqCOO (https://genentech.github.io/gneSeqCOO/) based on bulk tumor RNA sequencing [19], to classify the 29 DLBCL cell lines by COO, using the batch-corrected raw counts.

The double-hit/dark zone signature (DHIT/DZsig) classification was performed following the approach described by Ennishi *et al*. 20 and adapted for variance-stabilized transformation (VST) counts as described by Stokes *et al.*[21]. Briefly, 103 genes from the original 104-gene signature were Z-score transformed (mean = 0, standard deviation = 1). Gene-specific importance scores

were then combined with normalized expression values to calculate the final DHIT/DZsig score for each sample.

The DHIT/DZsig-positive threshold was defined by the last positive DHIT/DZsig-score sample with concurrent *BCL2* and *MYC* translocations; the DHIT/DZsig-negative threshold was defined by the last sample with a negative DHIT/DZsig score result, a positive mean Z-score of the negative genes and a negative mean Z-score of the positive genes. The remaining samples were classified as DHIT/DZsig-intermediate. Anchoring DHIT/DZ thresholds to double-hit status aligns with prior work and enhances biological specificity [20,22].

Shiny app development

An interactive Shiny application (DLBCL Cell Line Explorer) was developed in R (v4.5.2, www.R-project.org/) and RStudio (www.posit.co/) to enable systematic exploration of the molecular and transcriptomic features of the DLBCL cell line cohort analyzed in this study. The application integrates curated genomic, cytogenetic and transcriptomic annotations generated from the different analytical pipelines described before and provides an interactive framework for comparative interrogation of the dataset.

The platform incorporates information on genetic subtype assignment (including 2-S, LymphGen and DLBclass classifiers), COO (including Hans and gneSeqCOO), DHIT/DZsig classification, FISH-defined rearrangements (*MYC*, *BCL2* and *BCL6*) and somatic mutational profiles derived from targeted DNA sequencing. The interface allows dynamic filtering, visualization and comparison of individual cell lines and molecular subgroups, facilitating integrative analysis of lymphoma-associated molecular programs.

Gene expression analyses

Gene-set enrichment analyses (GSEA) were conducted using GSEA software v4.3.3 (Broad Institute, Cambridge, Massachusetts, USA) with DESeq2 library-size normalized counts as input.

GSEA was run using Signal2Noise analyses based on gene-set permutation. We used a custom-curated Lymphoma-Enriched gene set that contains selected gene sets, pathways related to lymphoid cells and lymphoma processes from different sources (**Table S3**), mainly Biocarta and other publicly available Molecular Signatures Databases (MsigDB)[23], combined with the gene sets described in Wright *et al.* [17], and the GSEA Hallmarks gene set collection. Significant pathways were considered when the FDR q-value was ≤ 0.25. Visualization of the GSEA results was carried out with ggplot2 v3.5.1 (RRID:SCR_014601), clusterProfiler v4.14.4 (RRID:SCR_016884), enrichplot v1.24.4 (RRID: SCR_018931).

Single sample GSEA (ssGSEA) projection was done using ssGSEA (v10.1) in the GSEA module in GenePattern (Broad Institute) with the combined custom-curated Lymphoma and Wright gene set database. For hierarchical clustering, ssGSEA projection (sample scores per pathway) was performed using Euclidean distance and the complete-linkage method in ComplexHeatmap v2.22.0 (RRID: SCR_017270). The differences between groups after ssGSEA were assessed using the Kruskal-Wallis test, followed by Dunn's post hoc multiple-comparison test. A p-value of < 0.05 was considered significant. Analyses were performed using GraphPad Prism 10 (Dotmatics) and R software v4.4.1.

Transcription factor (TF) activity scores were estimated using decouple R v2.12 and the DoRothEA database with a linear modeling approach, and TF enrichment comparisons between groups were performed using statistical tests from DESeq2. Only regulons (defined as sets of genes regulated as a unit) with confidence levels A or B and containing at least 5 target genes were included in the study. Confidence levels referred to the amount and quality of supporting evidence for each TF-target interaction. Levels A and B are supported by multiple curated resources or expert-reviewed literature, curated proof and/or ChIP-seq data (https://saezlab.github.io/dorothea/articles/dorothea.html)[24]. A p-value of < 0.05 was considered significant. Significant TFs inferred from the regulon analysis and differentially expressed genes (DEGs) identified by DESeq2 were integrated and visualized as a regulatory network using prior knowledge from DoRothEA [53]. The network included only interactions involving TFs with p-values ≤ 0.05, as well as DEGs with adjusted p-values ≤ 0.05 and absolute $\log_2$ fold changes≥ 1.

REFERENCES

1. Garcia M, Juhos S, Larsson M, et al. Sarek: A portable workflow for whole-genome sequencing analysis of germline and somatic variants. *F1000Res*. 2020;9:63. doi:10.12688/f1000research.16665.2

2. Hanssen F, Garcia MU, Folkersen L, et al. Scalable and efficient DNA sequencing analysis on different compute infrastructures aiding variant discovery. *NAR Genomics and Bioinformatics*. 2024;6(2):lqae031. doi:10.1093/nargab/lqae031

3. Di Tommaso P, Chatzou M, Floden EW, Barja PP, Palumbo E, Notredame C. Nextflow enables reproducible computational workflows. *Nat Biotechnol*. 2017;35(4):316-319. doi:10.1038/nbt.3820

4. Chen S, Zhou Y, Chen Y, Gu J. fastp: an ultra-fast all-in-one FASTQ preprocessor. *Bioinformatics*. 2018;34(17):i884-i890. doi:10.1093/bioinformatics/bty560

5. Li H. Aligning sequence reads, clone sequences and assembly contigs with BWA-MEM. *arXiv*. Preprint posted online May 26, 2013. doi:10.48550/arXiv.1303.3997

6. Benjamin D, Sato T, Cibulskis K, Getz G, Stewart C, Lichtenstein L. Calling Somatic SNVs and Indels with Mutect2. *bioRxiv*. Preprint posted online December 2, 2019:861054. doi:10.1101/861054

7. Pedersen BS, Quinlan AR. Mosdepth: quick coverage calculation for genomes and exomes. *Bioinformatics*. 2018;34(5):867-868. doi:10.1093/bioinformatics/btx699

8. Danecek P, Auton A, Abecasis G, et al. The variant call format and VCFtools. *Bioinformatics*. 2011;27(15):2156-2158. doi:10.1093/bioinformatics/btr330

9. Li H. A statistical framework for SNP calling, mutation discovery, association mapping and population genetical parameter estimation from sequencing data. *Bioinformatics*. 2011;27(21):2987-2993. doi:10.1093/bioinformatics/btr509

10. Wang K, Li M, Hakonarson H. ANNOVAR: functional annotation of genetic variants from high-throughput sequencing data. *Nucleic Acids Research*. 2010;38(16):e164. doi:10.1093/nar/gkq603

11. Tate JG, Bamford S, Jubb HC, et al. COSMIC: the Catalogue Of Somatic Mutations In Cancer. *Nucleic Acids Res*. 2019;47(D1):D941-D947. doi:10.1093/nar/gky1015

12. Bala Tannan N, Manzari MT, Herviou L, et al. Tumor-targeted nanoparticles improve the therapeutic index of BCL2 and MCL1 dual inhibition. *Blood*. 2021;137(15):2057-2069. doi:10.1182/blood.2020008017

13. Ghandi M, Huang FW, Jané-Valbuena J, et al. Next-generation characterisation of the Cancer Cell Line Encyclopedia. *Nature*. 2019;569(7757):503-508. doi:10.1038/s41586-019-1186-3

14. Morin RD, Mungall K, Pleasance E, et al. Mutational and structural analysis of diffuse large B-cell lymphoma using whole-genome sequencing. *Blood*. 2013;122(7):1256-1265. doi:10.1182/blood-2013-02-483727

15. Tarantelli C, Wald D, Munz N, et al. Targeting CD19-positive lymphomas with the antibodydrug conjugate loncastuximab tesirine: preclinical evidence of activity as a single agent and in combination therapy. *Haematologica*. 2024;109(10):3314-3326. doi:10.3324/haematol.2023.284197

16. Pedrosa L, Fernández-Miranda I, Pérez-Callejo D, et al. Proposal and validation of a method to classify genetic subtypes of diffuse large B cell lymphoma. *Scientific Reports*. 2021;11(1):1886. doi:10.1038/s41598-020-80376-0

17. Wright GW, Huang DW, Phelan JD, et al. A Probabilistic Classification Tool for Genetic Subtypes of Diffuse Large B Cell Lymphoma with Therapeutic Implications. *Cancer Cell*. 2020;37(4):551-568.e14. doi:10.1016/j.ccell.2020.03.015

18. Chapuy B, Wood T, Stewart C, et al. DLBclass: a probabilistic molecular classifier to guide clinical investigation and practice in diffuse large B-cell lymphoma. *Blood*. 2025;145(18):2041-2055. doi:10.1182/blood.2024025652

19. Harris W, Cao Y, Morschhauser F, et al. gneSeqCOO: a novel method for classifying diffuse large B-cell lymphoma cell of origin based on bulk tumor RNA sequencing profiles. *Leukemia & Lymphoma*. 0(0):1-8. doi:10.1080/10428194.2024.2446613

20. Ennishi D, Jiang A, Boyle M, et al. Double-Hit Gene Expression Signature Defines a Distinct Subgroup of Germinal Center B-Cell-Like Diffuse Large B-Cell Lymphoma. *Journal of Clinical Oncology*. 2019;37(3):190-201. doi:10.1200/jco.18.01583

21. Stokes ME, Wenzl K, Huang CC, et al. Transcriptomic classification of diffuse large B-cell lymphoma identifies a high-risk activated B-cell-like subpopulation with targetable MYC dysregulation. *Nat Commun*. 2024;15(1):6790. doi:10.1038/s41467-024-50830-y

22. Chan WC. Using Gene Expression Profiling to Move Beyond MYC/BCL2 Rearrangements in High-Grade Lymphoma. *J Clin Oncol*. 2019;37(3):175-177. doi:10.1200/JCO.18.01910

23. Arribas AJ, Campos-Martín Y, Gómez-Abad C, et al. Nodal marginal zone lymphoma: gene expression and miRNA profiling identify diagnostic markers and potential therapeutic targets. *Blood*. 2012;119(3):e9-e21. doi:10.1182/blood-2011-02-339556

24. Garcia-Alonso L, Holland CH, Ibrahim MM, Turei D, Saez-Rodriguez J. Benchmark and integration of resources for the estimation of human transcription factor activities. *Genome Res*. 2019;29(8):1363-1375. doi:10.1101/gr.240663.118
