# Supplementary material for: Decoding the Heterogeneity of Diffuse Large B‐Cell Lymphomas: A Comprehensive Genetic, Transcriptomic, and Phenotypic Profiling of B‐Cell Lymphoma Cell Lines
Source: Hematol Oncol. 2026 Aug 5;44(5):e70228. doi: 10.1002/hon.70228 (PMC13440701; doi:10.1002/hon.70228)
Supplement: Supplementary file 2 [file HON-44-e70228-s003.pdf]

Supplemental Figures for

Decoding the Heterogeneity of Diffuse Large B-Cell Lymphomas: A Comprehensive Genetic, Transcriptomic, and Phenotypic Profiling of B-Cell Lymphoma Cell Lines

Marina Pérez-Aguilera[1*], Lucía Pedrosa[1*], Sagrario Gómez[1], Sara Salazar-Ortego[2], Ismael Fernández-Miranda[1], Rafael Muñoz-Viana[3], Paloma Martín-Acosta[4], Beatriz L. Martín-Lunas[2], Rebeca Jimeno[2], Natalia Yanguas-Casás[1,5], Beatriz Horcajo[1], Giovana Roncador[6], José A. Martínez-Climent[7], Ana Ortega-Molina[2#], and Margarita Sánchez-Beato[1#].

[1]Lymphoma Research Group, Medical Oncology Department, Hospital Universitario Puerta de Hierro-Majadahonda, IDIPHISA, Madrid, Spain

[2]Metabolism in Cancer and Aging Research Group; Immune System Development and Function Department, Severo Ochoa Centre for Molecular Biology, CSIC-UAM, Madrid, Spain

[3]Bioinformatics Unit, Hospital Universitario Puerta de Hierro-Majadahonda, IDIPHISA, Madrid, Spain

[4]Cancer Molecular Pathology Group, Pathology Department, Hospital Puerta de Hierro-Majadahonda, IDIPHISA, Madrid, Spain

[5]Centro de Investigación Biomédica en Red de Fragilidad y Envejecimiento Saludable (CIBERFES), Madrid, Spain.

[6]Monoclonal Antibodies Core Unit Biotechnology Program Spanish National Cancer Research Centre (CNIO), Madrid, Spain.

[7]Department of Haematology, Center for Applied Medical Research, Cancer Center Clinica Universidad de Navarra, IDISNA, CIBERONC, Pamplona, Spain

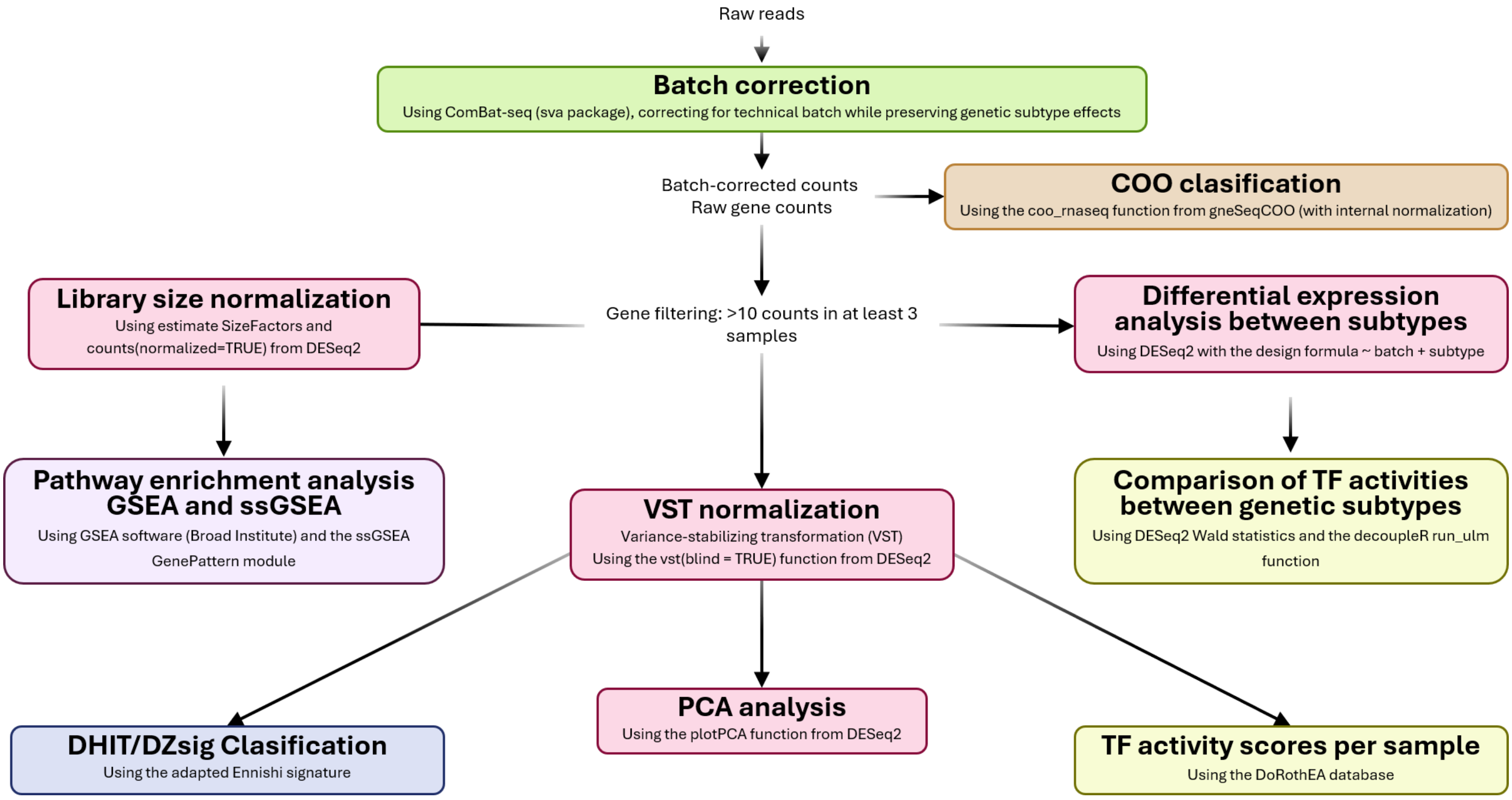

Figure S1: Schematic overview of the RNA-sequencing analytical workflow used in this study. Raw RNA-seq reads were processed to generate gene-level count matrices, followed by batch correction using ComBat-seq while preserving genetic subtype-associated variation. The resulting matrices were subsequently used for distinct downstream analyses according to the specific requirements of each method. Raw gene counts were used for COO classification with gneSeqCOO and for differential expression analyses using DESeq2 with the design formula ~ batch + subtype. Batch-corrected counts were subjected to library size normalization and variance-stabilizing transformation (VST) using DESeq2. DESeq2-normalized counts were used for pathway enrichment analyses, including GSEA and ssGSEA, as well as transcription factor (TF) activity inference with DoRothEA/decoupleR. VST-transformed counts were used for PCA analyses, DHIT/DZsig classification, and TF activity visualization.

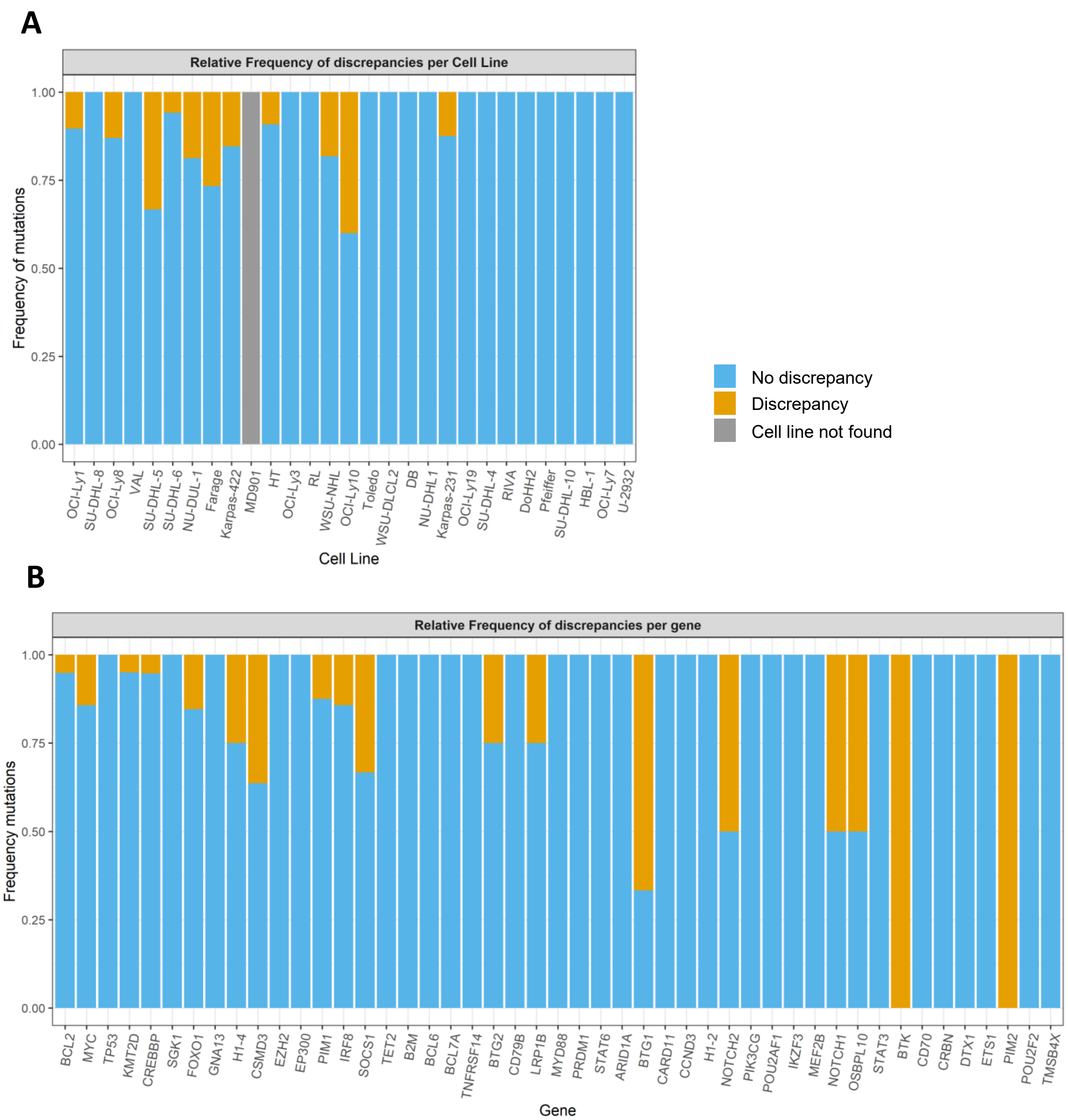

Figure S2. Discrepancies in detected mutations in diffuse large B-cell lymphoma cell lines. Relative frequency of discrepancies in detected mutations by DNA-targeted sequencing compared with external data, analyzed **A)** by cell line and **B)** by gene.

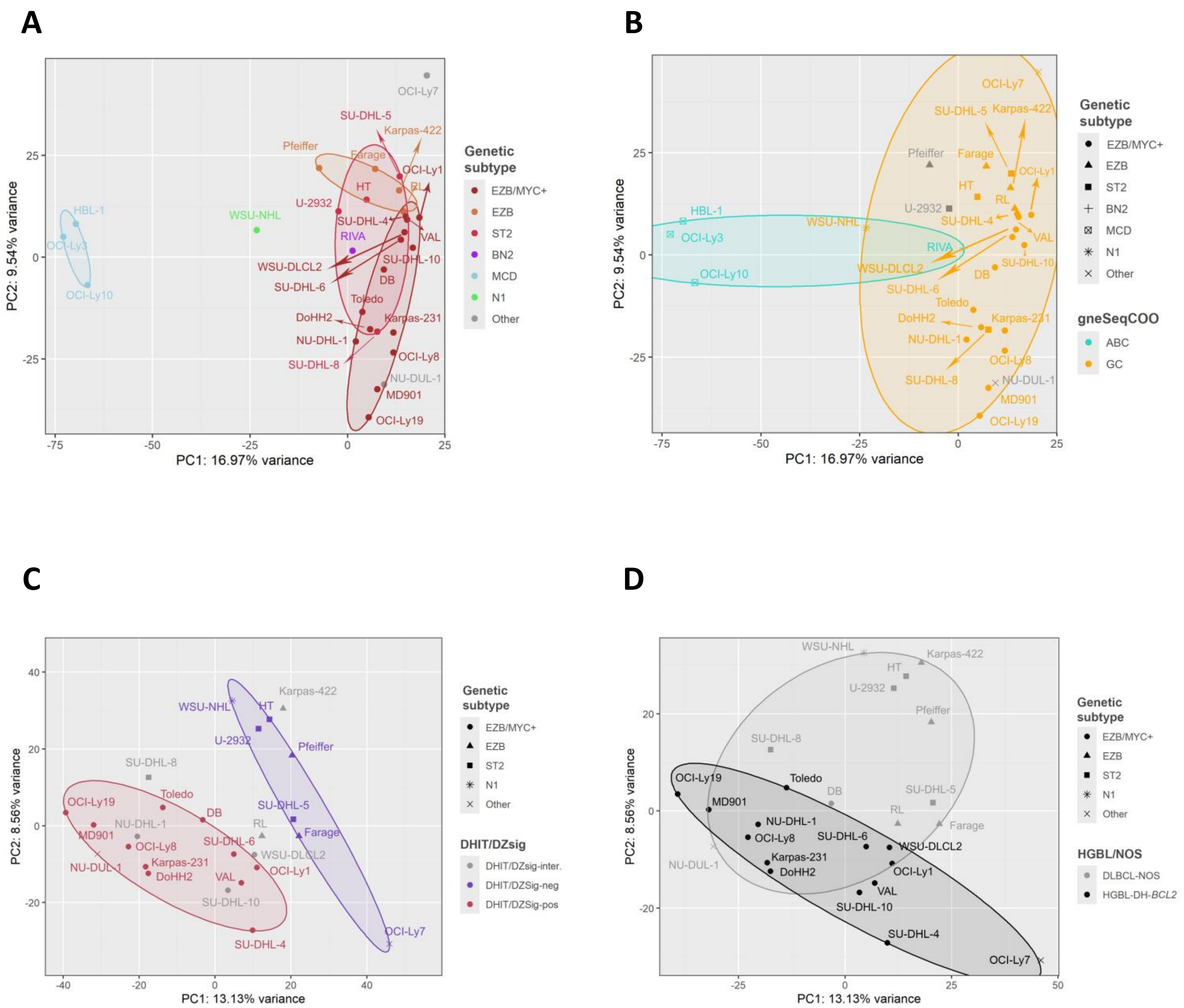

Figure S3. Principal component analysis (PCA). A) PCA of the 29 diffuse large B-cell lymphoma (DLBCL) cell lines according to the six genetic subgroups: EZB/MYC+, EZB, ST2, BN2, MCD, and N1. **B)** PCA according to cell-of-origin (COO) classification using the gnsSeqCOO method: activated B-cell-like (ABC), germinal center B-cell-like (GC), or unclassified. **C)** PCA of 25 DLBCL cell lines, excluding ABC cell lines, according to DHIT/DZsig status. **D)** PCA according to the classification as DLBCL, not otherwise specified (DLBCL-NOS), or high-grade B-cell lymphoma double hit with *MYC* and *BCL2* rearrangements (HGBL-DH-*BCL2*).

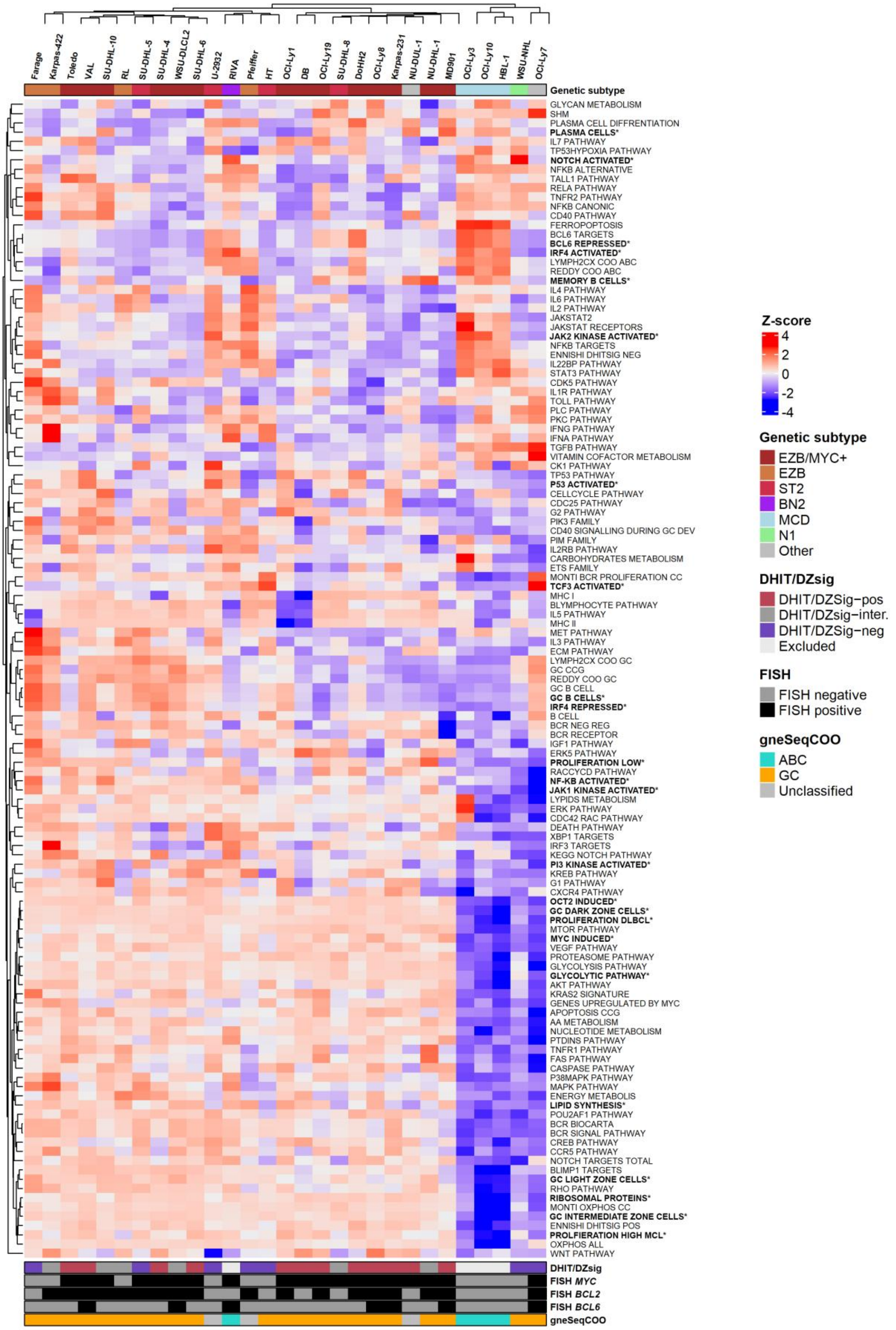

Figure S4. Pathway Enrichment Analysis in diffuse large B-cell lymphoma cell lines based on genetic subtype. Single-sample Gene Set Enrichment Analysis (ssGSEA) was performed to assess key pathways associated with lymphoma, and the results are visualized as a heatmap. DLBCL cell lines are categorized by unsupervised hierarchical clustering. The figure also displays the double-hit/dark zone signature (DHIT/DZsig) score for each cell line, the corresponding genetic subtype determined by 2-S algorithm, the translocations of *MYC*, *BCL2*, and *BCL6* genes, and the cell-of-origin (COO) classifications determined by gneSeqCOO (activated B-cells [ABC], germinal center B-cells [GC] or unclassified). Gene sets from Wright et al.[14] are indicated in bold and with an asterisk (*).

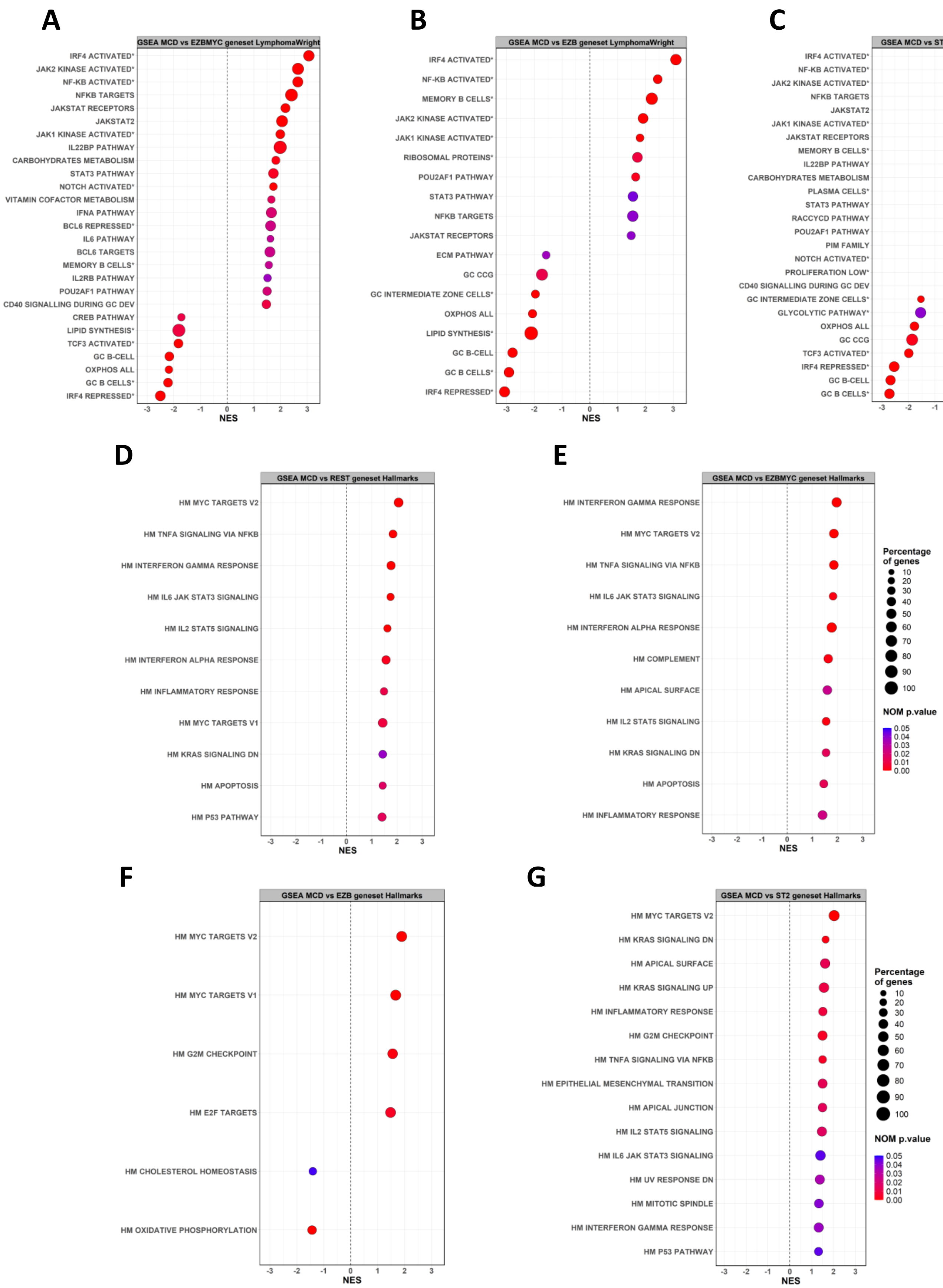

Figure S5. Gene Set Enrichment Analysis in MCD-derived cell lines. A-C) Normalized enrichment score (NES) of custom-curated Lymphoma-Enriched gene sets when comparing gene expression in MCD subtype versus **A)** EZB/MYC+ subtype; **B)** EZB subtype; **C)** ST2 subtype**. D-G)** NES of Hallmarks gene sets when comparing gene expression in MCD subtype versus **D)** the rest of diffuse large B-cell lymphoma cell lines; **E)** EZBMYC+ subtype; **F)** EZB subtype, and **G)** ST2 subtype.

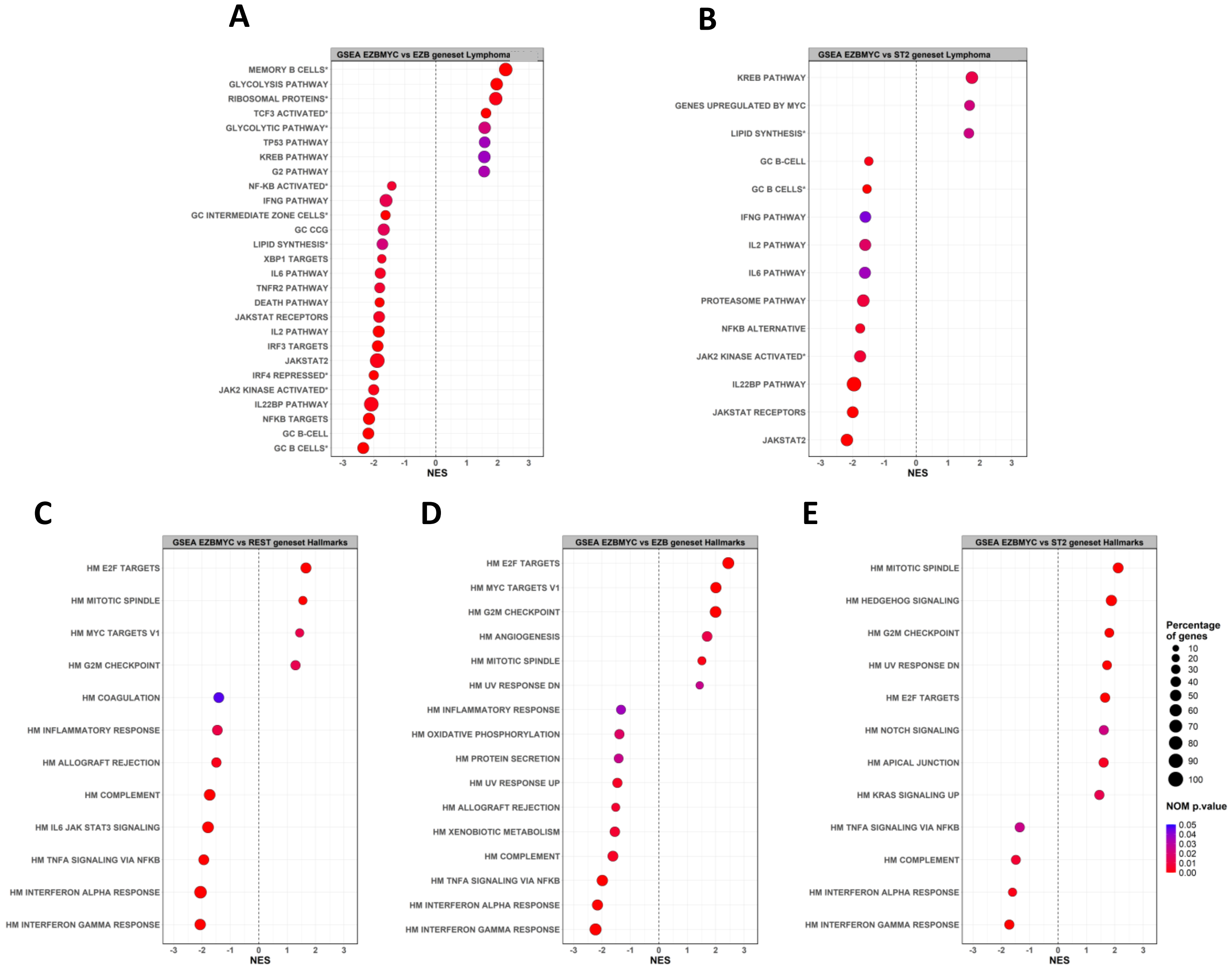

Figure S6. Gene Set Enrichment Analysis in EZBMYC+-derived cell lines. **A-B)** Normalized enrichment score (NES) of custom-curated Lymphoma-Enriched gene sets when comparing gene expression in EZB/MYC+ subtype versus **A)** EZB subtype, and **B)** ST2 subtype. **C-E)** NES of Hallmarks gene sets when comparing gene expression in EZB/MYC+ subtype versus **C)** the rest of the diffuse large B-cell lymphoma cell lines; **D)** EZB subtype; and **E)** ST2 subtype.

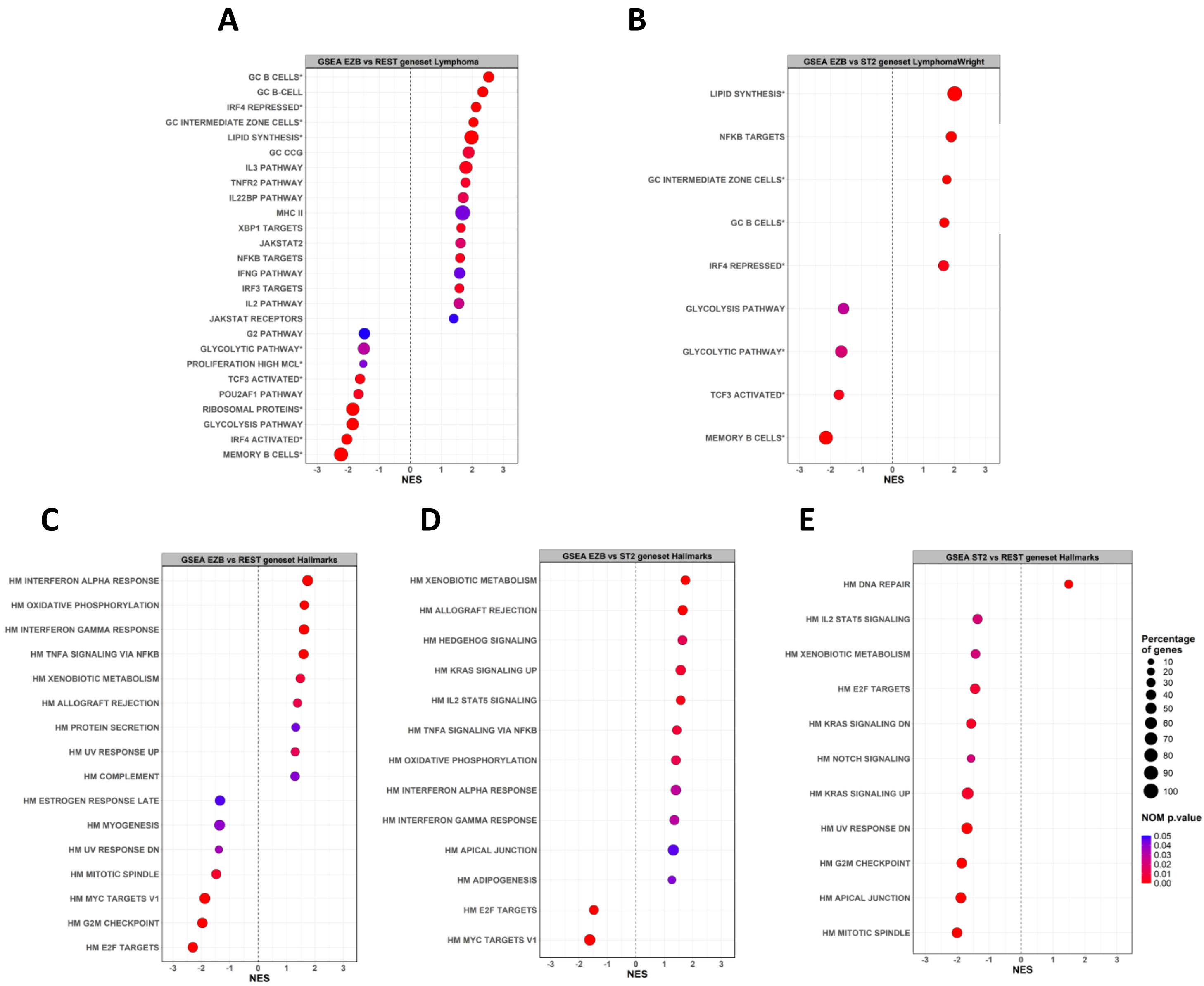

Figure S7. Gene Set Enrichment Analysis in EZB and ST2-derived cell lines. A-B) Normalized enrichment score (NES) of custom-curated Lymphoma-Enriched gene sets when comparing gene expression in EZB subtype versus **A)** the rest of the diffuse large B-cell lymphoma (DLBCL) cell lines; and **B)** ST2 subtype. **C-D)** NES of Hallmarks gene sets when comparing gene expression in EZB subtype versus **C)** the rest of the DLBCL-derived cell lines; and **D)** ST2 subtype. **E)** NES of Hallmarks gene sets when comparing gene expression in ST2 subtype versus the rest of the DLBCL-derived cell lines.

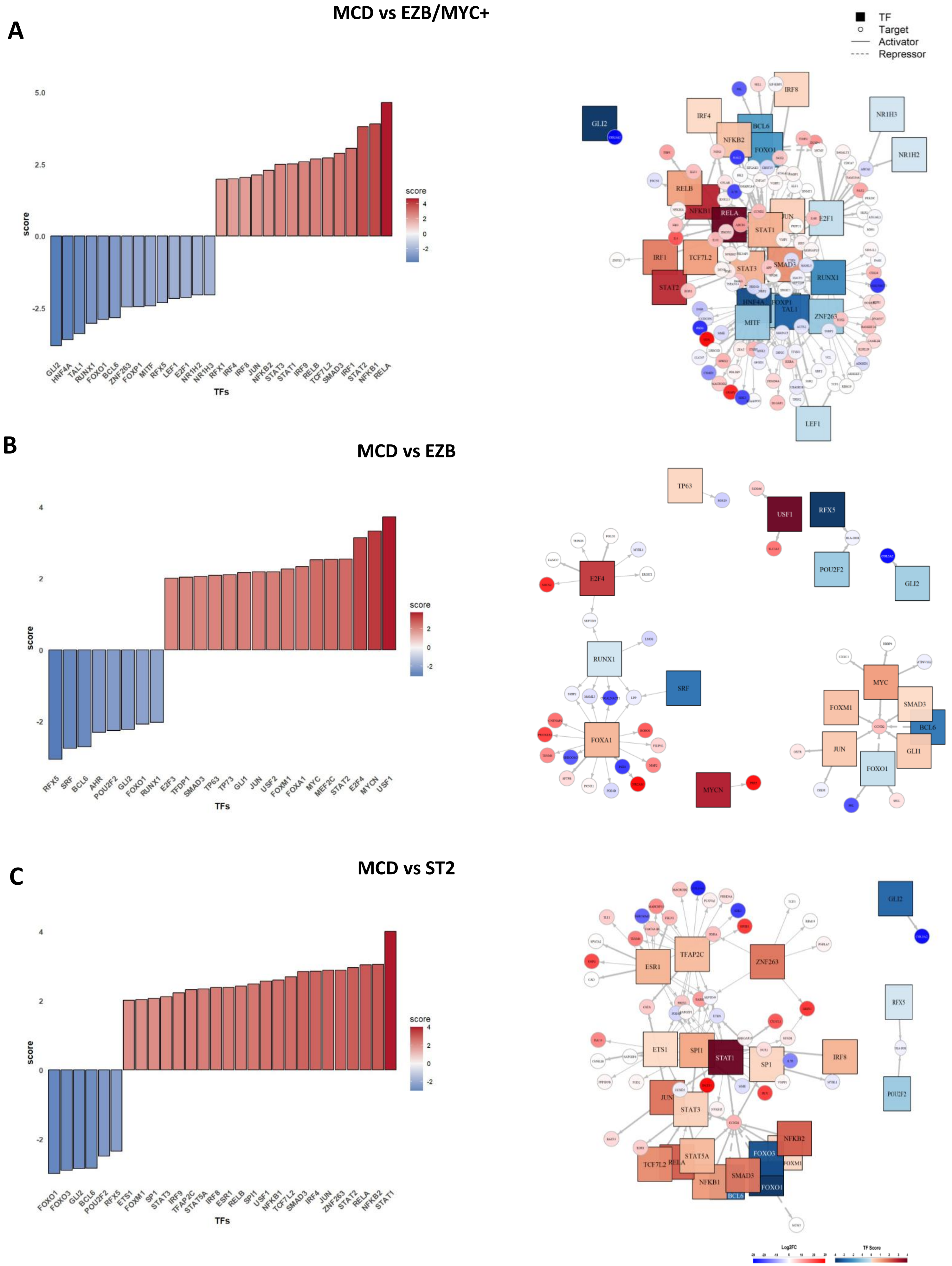

Figure S8. Regulatory Network of Significantly Active Transcription Factors (TFs) when comparing TFs activity in **A)** MCD vs EZB/MYC+ subtype; **B)** MCD vs EZB subtype; and **C)** MCD vs ST2 subtype. Left: Inferred TF activity score; Right: Network plots for each comparison

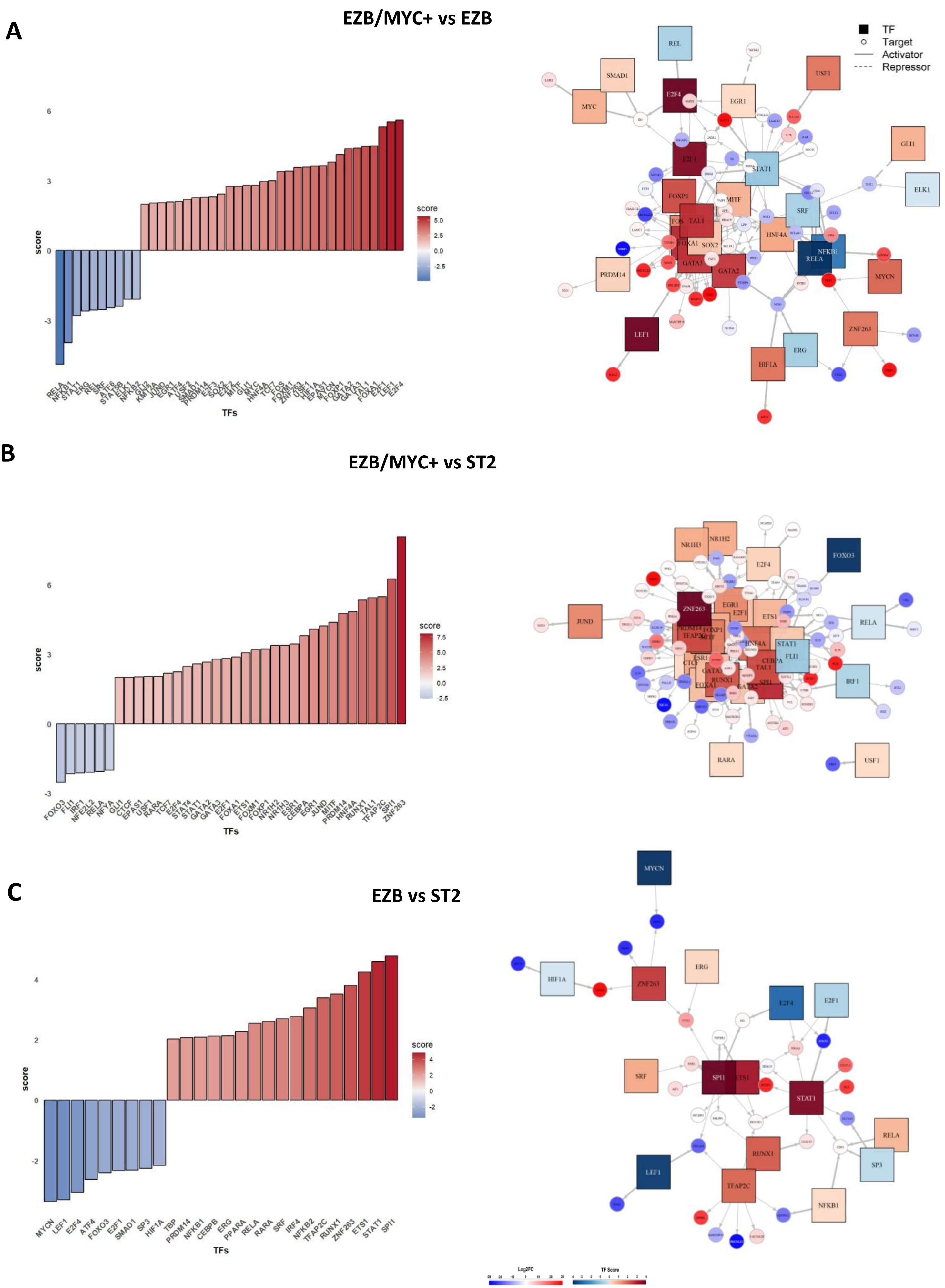

Figure S9. Regulatory Network of Significantly Active Transcription Factors (TFs) when comparing TFs activity in **A)** EZB/MYC+ vs EZB subtype; **B)** EZB/MYC+ vs ST2 subtype; and **C)** ST2 vs EZB subtype. Left: Inferred TF activity score. Right: Network plots for each comparison
